# Venom-gland transcriptome and venom proteome of the Malaysian king cobra (*Ophiophagus hannah*)

**DOI:** 10.1186/s12864-015-1828-2

**Published:** 2015-09-10

**Authors:** Choo Hock Tan, Kae Yi Tan, Shin Yee Fung, Nget Hong Tan

**Affiliations:** Department of Pharmacology, Faculty of Medicine, University of Malaya, Kuala Lumpur, 50603 Malaysia; Department of Molecular Medicine, Faculty of Medicine, University of Malaya, Kuala Lumpur, 50603 Malaysia

**Keywords:** *Ophiophagus hannah*, King cobra, Venom proteome, Venom gland transcriptome, Nano-LCMS, ESI-LCMS/MS

## Abstract

**Background:**

The king cobra (*Ophiophagus hannah*) is widely distributed throughout many parts of Asia. This study aims to investigate the complexity of Malaysian *Ophiophagus hannah* (MOh) venom for a better understanding of king cobra venom variation and its envenoming pathophysiology. The venom gland transcriptome was investigated using the Illumina HiSeq™ platform, while the venom proteome was profiled by 1D-SDS-PAGE-nano-ESI-LCMS/MS.

**Results:**

Transcriptomic results reveal high redundancy of toxin transcripts (3357.36 FPKM/transcript) despite small cluster numbers, implying gene duplication and diversification within restricted protein families. Among the 23 toxin families identified, three-finger toxins (3FTxs) and snake-venom metalloproteases (SVMPs) have the most diverse isoforms. These 2 toxin families are also the most abundantly transcribed, followed in descending order by phospholipases A_2_ (PLA_2_s), cysteine-rich secretory proteins (CRISPs), Kunitz-type inhibitors (KUNs), and L-amino acid oxidases (LAAOs). Seventeen toxin families exhibited low mRNA expression, including hyaluronidase, DPP-IV and 5’-nucleotidase that were not previously reported in the venom-gland transcriptome of a Balinese *O. hannah*. On the other hand, the MOh proteome includes 3FTxs, the most abundantly expressed proteins in the venom (43 % toxin sbundance). Within this toxin family, there are 6 long-chain, 5 short-chain and 2 non-conventional 3FTx. Neurotoxins comprise the major 3FTxs in the MOh venom, consistent with rapid neuromuscular paralysis reported in systemic envenoming. The presence of toxic enzymes such as LAAOs, SVMPs and PLA_2_ would explain tissue inflammation and necrotising destruction in local envenoming. Dissimilarities in the subtypes and sequences between the neurotoxins of MOh and *Naja kaouthia* (monocled cobra) are in agreement with the poor cross-neutralization activity of *N. kaouthia* antivenom used against MOh venom. Besides, the presence of cobra venom factor, nerve growth factors, phosphodiesterase, 5’-nucleotidase, and DPP-IV in the venom proteome suggests its probable hypotensive action in subduing prey.

**Conclusion:**

This study reports the diversity and abundance of toxins in the venom of the Malaysian king cobra (MOh). The results correlate with the pathophysiological actions of MOh venom, and dispute the use of *Naja* cobra antivenoms to treat MOh envenomation. The findings also provide a deeper insight into venom variations due to geography, which is crucial for the development of a useful pan-regional antivenom.

**Electronic supplementary material:**

The online version of this article (doi:10.1186/s12864-015-1828-2) contains supplementary material, which is available to authorized users.

## Background

Toxin proteins appeared more than 200 million years ago in the common ancestor of venomous reptiles such as snakes and their saurian cousins [1]. The prevailing thoughts on venom evolution agree that toxin genes were initially recruited from genes of proteins with normal physiological functions, followed by repeated gene duplication that creates redundancy, allowing a gene copy to be selectively expressed in the venom gland. The ‘free’ copies subsequently underwent neofunctionalization through positive selection and molecular adaptation at accelerated rates, driven primarily by changes in the ecological niche, diet and predator–prey arms race [2]. Besides gene duplications, alternative splicing and alterations of domain sturctures are other mechanisms capable of generating novel toxin genes [3]. The emergence of paralogous groups of multigene families across taxonomic lineages is accompanied by the occurrence of multiple isoforms within each major toxin family, resulting in diverse functional variations of venom proteins that have great impacts on medical management and toxinological research [4]. The immerse variety of toxins also serves as a source of leads for drug discovery. From a clinical standpoint, the phenomenon gives rise to diverse snakebite syndromes and potentially suboptimal antivenom effectiveness. The phenomenon complicates snakebite management that relies heavily on the understanding of venom complexity and the availability of effective antivenom.

In most developing and under-developed countries, snake envenomation remains a neglected tropical disease and a disease of poverty [5]. Malaysia, as a tropical country that is home to many venomous snake species, shares similar public health concerns. The problem affects not only the rural populations but also the suburban regions due to rapid urbanization as the human populations increasingly encroach upon snake habitat [6–8]. Among the native snakes in Malaysia, the king cobra (*Ophiophagus hannah*) is a known cause of envenomation [6, 7], partly due to its popularity among snake hobbyists. The king cobra is the world’s largest venomous snake (over 3 m in length), capable of delivering huge amounts of venom per bite [9] (in our experience, > 1 g of dry venom mass per milking from this adult king cobra). Clinically king cobra bites cause neurotoxicty, paralysing respiratory muscles and leading to rapid death if untreated. In surviving victims, the bites can result in extensive tissue necrosis and crippling deformity, adding to the heavy toll of human suffering in socioeconomically deprived countries.

*Ophiophagus hannah* is widely distributed across the Indian subcontinent, southeastern Asia and the southern part of China [9]. As the generic name *Ophiophagus* (Greek: *Ophio*-snakes, -*phagous*-eating) suggests, its diet consists primarily of snakes, although small mammals are also eaten. The *Ophiophagus* is a monotypic genus with only one species recognized currently. However, its vast geographical distribution accompanied by morphological differences suggests potential taxonomic divergence and variation in venom composition. In fact, the venom of king cobra has been studied since the early 1970’s. Various components have been isolated and characterized, including L-amino acid oxidases [10], metalloproteinases [11], three finger toxins (3FTxs) [12, 13], phospholipases A_2_ (PLA_2_s) [14, 15], ohanin [16], kunitz-type protease inhibitors [17] and factor X activator [18]. Nonetheless, the significance of the origin and intraspecific variation of king cobra venom has only been recently documented. In a study with venom samples sourced from a few countries, differences in venom properties were noted between Chinese and Southeast Asian king cobras [19]. A genomic study of an Indonesian king cobra (a Balinese specimen) by Vonk et al. [20] indicated great diversity in the PLA_2_ and 3FTx gene families in a single specimen. Indeed, intraspecific variations of snake venom are attributed to multiple factors such as diet, geographical distribution, ontogeny, season/climate changes etc. [21], and the phenomenon is hence highly relevant to the king cobra in view of its extremely vast distribution. Producing regional or local antivenom would be ideal; unfortunately the production of an affordable and effective antivenom tailored to different areas by local manufacturers would be hampered by financial and technical constraints. Thus, a new approach has been recently proposed by the toxinological community to focus on the investigation of specific venom-gland transcriptomes and venom proteomes for the development of pan-regional antivenoms [22, 23].

Vonk et al. [20] reported the venom-gland transcriptome and venom proteome of a specimen from the Indonesian island of Bali. However, the possibility of intraspecific venom variations from king cobra in the distant Malayan Peninsula cannot be excluded. In addition, the paper reported the numbers of toxin subtypes identified in the venom proteome without the relative toxin abundances. Earlier, Vejayan et al. [24] reported 2-dimensional gel electrophoresis findings of the Malaysian king cobra venom (commercial source, unspecified exact locality of the snake). Unfortunately, their mass spectrometric analysis (MALDI-TOF MS) yielded a large number of unidentifiable proteins, whilst only five toxin protein families were determined (PLA_2_s, 3FTxs, serine protease inhibitors, complement depleting factors, and ohanin) as well as one non-toxin protein, thioredoxin. The study verified the presence of some previously isolated toxins but shed little light on the venom proteome. Another recent report on king cobra(s) venom of probable Thai origin profiled the venom as well, detecting 14 protein types/families (including hypothetical proteins), but again without the estimation of relative toxin abundance [25]. The 1D electrophoretic gel findings of the Thai king cobra venom appeared similar to that reported by Chang et al. [19], suggesting that 3FTxs are the major component of king cobra venoms of different localities. However, the diversity in toxin sequences and isoforms as well as variations in toxin abundance could not be ascertained. To remedy the current lack of venom-gland transcriptomic and venom proteomic data for the Malaysian king cobra, the current study employed next-generation sequencing technology (massively parallel sequencing) and nano-liquid chromatography-tandem mass spectrometry (nano-LCMS/MS) for a deeper insight into the toxin diversity of king cobra venom.

## Results

### Sequencing and transcriptome assembly

**Fig. 1:**
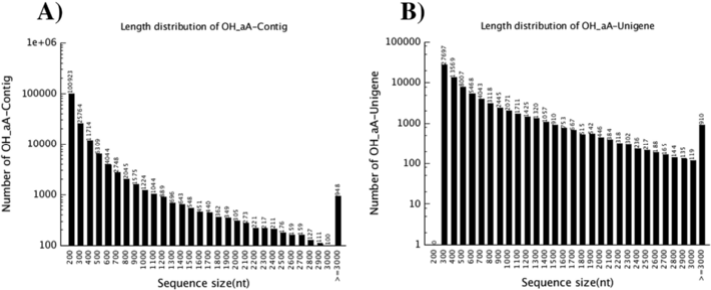
*De novo* transcriptome assembly with short reads assembling program, Trinity. **a** Length distribution of Contigs from reads assembly. **b** Length distribution of Unigenes generated from connected Contigs

**Fig. 2:**
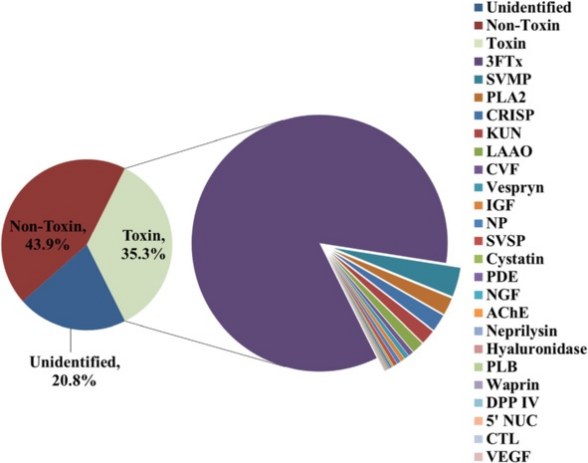
The Malaysian *Ophiophagus hannah* venom gland mRNA expression profile. Pie charts mainly display the percentage abundance of toxin transcripts. Toxins transcripts accounted for 35.3 % of the total FPKM. Three-finger toxins are the most abundant toxin family in the venom gland (84.9 % of all toxin transcripts). 3FTx, three-finger toxin; SVMP, snake venom metalloproteinase; PLA_2_, phospholipase A_2_; CRISP, cysteine-rich secretory protein; KUN, Kunitz-type proteinase inhibitor; LAAO, L-amino acid oxidase; CVF, Cobra venom factor; IGF, insulin-like growth factor; NP, natriuretic peptide; SVSP, snake venom serine protease; PDE, phosphodiesterase; NGF, nerve growth factor; AChE, acetylcholinesterase; PLB, phospholipase-B; DPP IV, dipeptidylpeptidase IV; CTL, C-type lectin; VEGF, vascular endothelial growth factor

From the primary output statistics of transcript sequencing, a total of 52,280,572 pairs of clean reads passed the Illumina quality filter for further *de novo* assembly using the short reads assembling program, Trinity [26]. Trinity created 164,775 Contigs (N50 = 459), connected to form 78,882 Unigenes (N50 = 864) in total as shown in Fig. 1. BLASTx alignment (e-value < 0.00001) between the Unigenes and sequences in the NCBI non-redundant (nr) protein database yielded 36,753 annotated Unigenes (Additional file 1). After filtering low-frequency transcripts (less than 1 FPKM, Fragments Per Kilobase of exon model per Million mapped reads), the assemblies were reduced from 78,882 to 68,472 transcripts and categorized into unidentified (39,888 transcripts), non-toxin (28,456) and toxin (128) groups. Of these, 20 toxin transcripts encode full-length toxin sequences. Although the toxin group accounted for 128 transcripts only, it is abundantly expressed as much as 35.3 % of the total expression (based on the parameter FPKM), whereas the unidentified and non-toxin groups were 20.8 % and 44.9 % respectively (Fig. 2). Toxin transcripts were expressed at extremely higher redundancies (3357.36 FPKM/transcript) compared to non-toxin transcripts (18.81 FPKM/transcript) (Additional file 1).

### Venom-gland transcriptome

The identifiable toxin transcripts (128 partial and complete transcripts, 35.3 % of the total FPKM) represent 23 protein families related to venom toxic functions. The toxin constituents, as indicated by FPKM, are dominated by 3FTxs (84.9 %). The toxin transcript abundances are followed by snake venom metalloproteases (SVMPs, 3.7 %), phospholipases A_2_ (PLA_2_s, 2.2 %), cysteine-rich secretory proteins (CRISPs, 2.1 %), Kunitz-type proteinase inhibitors (KUNs, 1.8 %) and L-amino acid oxidases (LAAOs, 1.5 %). A variety of toxin or protein mRNAs is expressed in low abundances (<1 %), including cobra venom factors (CVFs), vespryn, natriuretic peptides (NPs), snake venom serine proteases (SVSPs), phosphodiesterases (PDEs), nerve growth factors (NGFs), acetylcholinesterases (AChEs), neprilysins, hyaluronidase, phopholipases B (PLB), cystatins, dipeptidylpeptidase IV (DPP-IV), 5’nucleotidases (5’NUCs), waprins, C-type lectins (CTLs), insulin-like growth factors (IGFs), and vascular endothelial growth factors (VEGFs) (Fig. 2). The sequences and parameter details of the transcripts were sorted according to gene families and compiled in Additional file 2.

### Venom Proteome

**Fig. 3:**
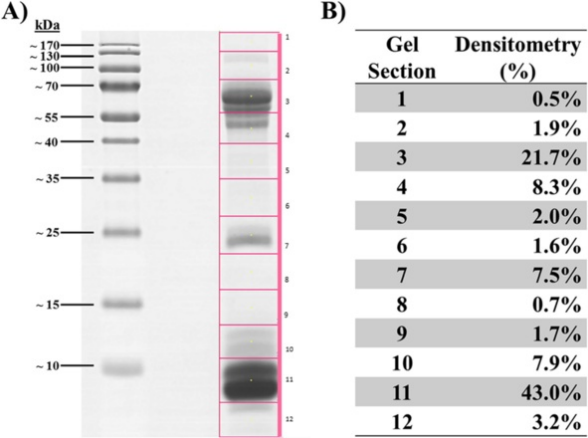
Densitometry quantitative measurement of SDS-PAGE gel using myImageAnalysis Software. **a** SDS-PAGE gel sections cutting scale with protein marker range from ~10kDa to 170kDa. **b** Gel densitometry section 1 to 12 displayed in percentages (%)

When examined under reducing 15 % SDS-PAGE, MOh venom showed three major protein bands each with a distinct molecular mass range: high (50-70 kDa), intermediate (20-30 kDa), and low (<14 kDa). The gel as shown in Fig. 3 was divided into 12 sections of equal sizes in preparation for in-gel digestion and nano-LCMS/MS, and the protein abundances from each section were estimated by gel densitometry for in-gel protein amount correlation [27]. Proteins were detected by nano-LCMS/MS from all gel sections including the pale-staining ones (Sections 1, 2, 5, 6, 8 and 9) notwithstanding their low abundances. The MOh venom proteins mainly migrated to Section 3, 4, 7, 10, 11 and 12, accounting for approximately 90 % (21.7 %, 8.3 %, 7.5 %, 7.9 %, 43.0 % and 3.2 %, respectively) of the total venom protein (Additional file 3).

**Fig. 4:**
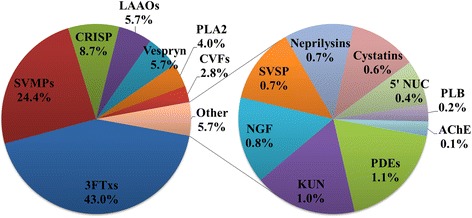
Protein expression profiles of Malaysian *O. hannah* venom with a total of 116 unique toxins peptides identified encoded for 16 protein families. Three-finger toxins are the most abundant toxin family in the MOh venom (44.01 %). 3FTx, three-finger toxin; LAAO, L-amino acid oxidase; SVMP, snake venom metalloproteinase; CRISP, cysteine-rich secretory protein; CVF, cobra venom factor; PLA_2_, phospholipase A_2_; 5’NUC, 5’nucleotidase; KUN, Kunitz-type protease inhibitor; SVSP, snake venom serine protease; PDE, phosphodiesterase; NGF, nerve growth factor; PLB, phospholipase-B; AChE, acetylcholinesterase

A customized reference database (NCBI Serpentes merged with MOh venom-gland transcriptome) was used in data mining to identify peptides sequenced by tandem mass spectrometry. A total of 45 toxins were identified from the MOh venom; 20 of these were matched to the transcripts. All toxins were assigned to 16 protein families. 3FTxs and SVMPs were the most abundantly expressed toxins in the proteome, accounting for 43.0 % and 24.4 %, respectively, of the venom. This was followed by CRISP (8.7 %), LAAOs (5.7 %), vespryn (5.7 %), PLA_2_ (4.0 %) and CVFs (2.8 %). Protein families with an abundance of less than 2 % each were categorized into “others” and considered as low-abundance proteins (a total of 9 protein families) (Fig. 4). The sequences and parameter details of the proteins identified were sorted according to gel sections and protein families as compiled in Additional file 3.

**Fig. 5:**
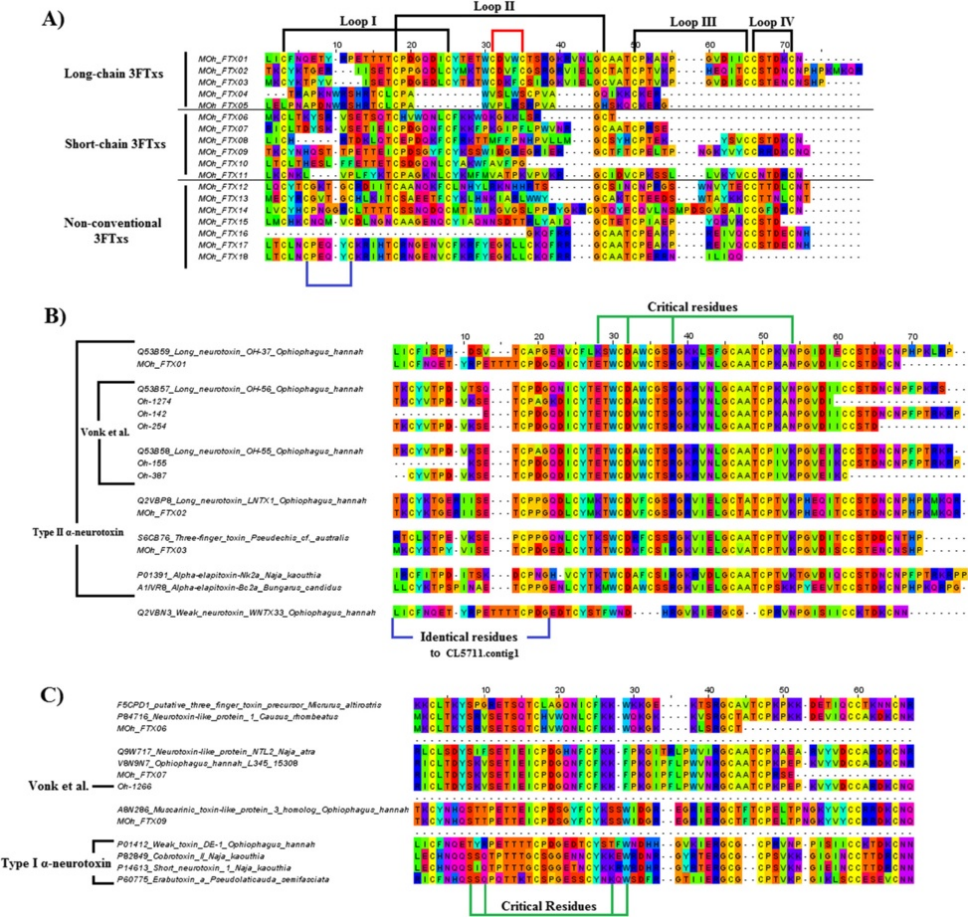
Sequence alignments of three-finger toxin (3FTx) transcripts of Malaysian *O. hannah* venom-gland transcriptome. **a** Malaysian *O. hannah* 3FTxs were aligned to show disulfide bonding (Black line: disulfide bond loops; Blue: additional disulfide bond of S-3FTxs; Red line: additional disulfide bond of L-3FTxs). **b** L-3FTxs were aligned and compared to sequences from Balinese king cobra [20] and public database (Green line: critical residues of L-3FTxs; Blue: identical residues to WNTX-33). **c** S-3FTxs were aligned and compared to sequences from Balinese king cobra [20] and and public database (Green: critical residues of S-3FTx)

**Fig. 6:**
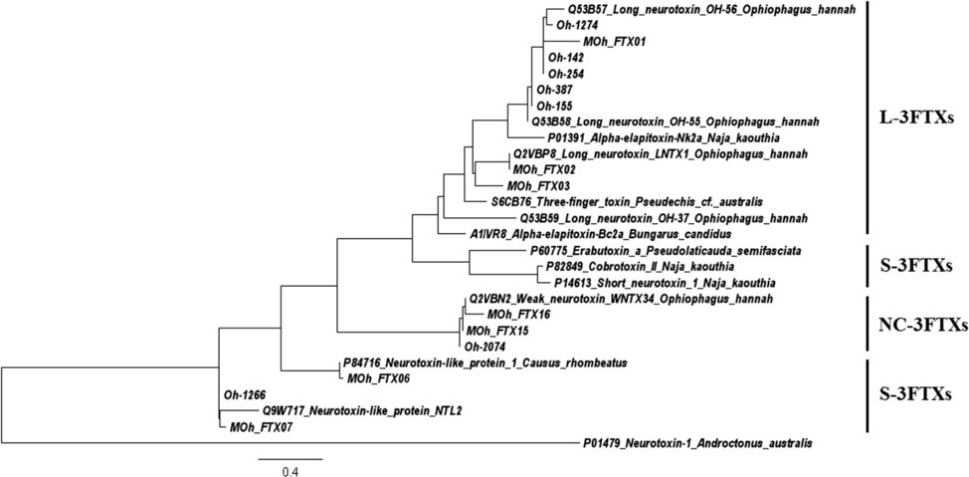
Phylogenetic tree of selected 3FTx transcripts of Malaysian *O. hannah* constructed with MEGA 6 using the Maximum likelihood method and presented by FigTree 1.4. (UniprotKB accession numbers of proteins used to construct the tree are as follows: Q53B59: Long neurotoxin OH-37, Q53B57: Long neurotoxin OH-56, Q53B58: Long neurotoxin OH-55, Q2VBP8: Long neurotoxin LNTX1, S6CB76: Three-finger toxin, P01391: Alpha-elapitoxin-Nk2a, A1IVR8: Alpha-elapitoxin-Bc2a, Q9W717: Neurotoxin-like protein NTL2, P84716: Neurotoxin-like protein 1, P82849: Cobrotoxin II, P14613: Short neurotoxin 1, P60775: Erabutoxin a, P01479: Neurotoxin-1). Neurotoxin-1 *Androctonus australis* used as outgroup

**Fig. 7:**
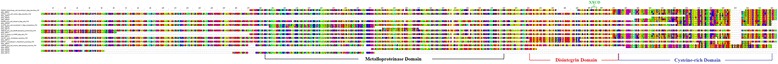
Sequence alignments of snake venom metalloproteinase (SVMP) transcripts of Malaysian *O. hannah* venom-gland transcriptome in comparison with SVMP sequences of representative venomous snakes. (Black: metalloproteinase domain; Red: disintegrins domain; Blue: cysteine-rich domain; Green: XXCD region)

**Fig. 8:**
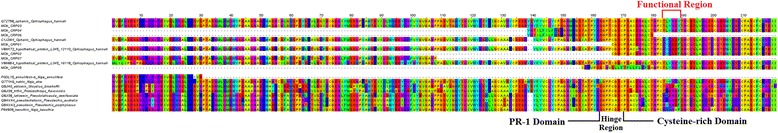
Sequence alignments of cysteine-rich secretory protein (CRISP) transcripts of Malaysian *O. hannah* venom-gland transcriptome in comparison with CRISP sequences of representative venomous snakes. (Black lines differentiate PR-1 domain/Hinge region/Cysteine-rich domain; Red: Functional region)

**Fig. 9:**
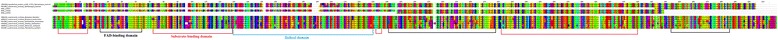
Sequence alignments of L-amino acid oxidase (LAAO) transcripts of Malaysian *O. hannah* venom-gland transcriptome in comparison with LAAO sequences of representative venomous snakes. (Black: FAD-binding domain; Red: substrate-binding domain; Blue: helical domain)

**Fig. 10:**
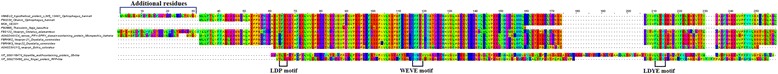
Sequence alignments of vespryn (ohanin) transcripts of Malayan *O. hannah* venom-gland transcriptome in comparison with vespryn sequences of representative venomous snakes. (Blue: additional residues possessed by hypothetical protein L345_13461; Black: three conserved LDP, WEVE, and LDYE motifs of B30.2-like domains containing protein)

**Fig. 11:**
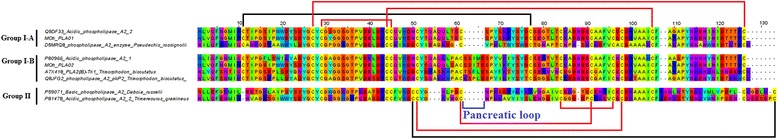
Sequence alignments of phospholipase A_2_ (PLA_2_) transcripts of Malaysian *O. hannah* venom-gland transcriptome in comparison with PLA_2_ sequences of representative venomous snakes. (Red lines: conservative disulfide bonds; Black lines: additional disulfide bond; Blue: 62-67 residues of pancreatic loop)

**Fig. 12:**
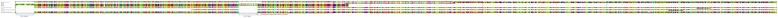
Sequence alignments of cobra venom factor (CVF) transcripts of Malaysian *O. hannah* venom gland transcriptome in comparison with CVF sequences of representative venomous snakes. (Black: alpha-chain; Red: gamma-chain; Green: beta-chain; Blue: extra regions)

Based on the proteomic data, transcript sequences of translated major toxins were selected for sequence comparison with related toxins. The sequence alignments of the major transcripts were illustrated in the following figures according to their respective toxin families: 3FTxs (Fig. 5), SVMPs (Fig. 7), CRISPs (Fig. 8), LAAOs (Fig. 9), vespryns (Fig. 10), PLA_2_s (Fig. 11) and CVFs (Fig. 12). Figure 6 shows the phylogenetic tree of the major 3FTxs of MOh.

### Comparison between transcriptome and proteome

In this study, data from the venom-gland transcriptome (FPKM metrics) and the venom proteome (cumulative of peptide fragment ratio per unit length) derived from the same specimen did not show significant correlation (FPKM v.s. PPUL metrics: r^2^ = 0.013, p > 0.05).

Table 1 summarized the quantitative results of the transcripts and proteins by toxin family for the Malaysian king cobra studied. In addition, the “–omic” information available for the Balinese king cobra [20] was included along in Table 1 for comparison purposes. Of note, the transcripts by protein family for the Balinese specimen were denoted as present or absent as quantitative data on transcript abundances were not available [20]. In addition, the expression of the percentage of toxin family in the venom proteome is different between the two studies: this current study (on Malaysian king cobra) reports the relative abundance of protein in each toxin family (in addition to the number of protein subtypes); whereas the other (on Balinese king cobra) [20] indicated the relative number of toxin subtypes without estimation of protein abundance. Sequences of toxin transcripts of the two king cobras were compared and compiled in Additional file 4.

**Table 1:** Comparison of the venom-gland transcriptomes and the venom proteomes of king cobra (*Ophiophagus hannah*, Oh) from Malaysia (Seremban) and Indonesia (Bali)

	Venom-gland transcriptome		Venom Proteome	
	Malaysian Oh (Current study)	Balinese Oh (Vonk et al., 2013)	Malaysian Oh (Current study)	Balinese Oh (Vonk et al., 2013)
Toxin Family	^a^Transcript (abundance in %)	^b^Transcript detected	^c^Relative abundance of toxin in % (number of subtype)	^d^Relative presence of toxin in % (number of subtype)
3FTXs	84.95	√	43.0 (13)	41.1 (30)
SVMP	3.68	√	24.4 (12)	24.7 (18)
PLA2	2.16	√	4.0 (1)	5.5 (4)
CRISP	2.12	√	8.7 (1)	6.8 (5)
KUNs	1.84	√	1.0 (1)	4.1 (3)
LAAO	1.47	√	5.7 (2)	1.4 (1)
CVF	0.63	√	2.8 (4)	5.5 (4)
Vespryn	0.58	√	5.7 (1)	1.4 (1)
IGF	0.52	√	-	-
NP	0.48	√	-	1.4 (1)
SVSP	0.47	√	0.7 (1)	4.1 (3)
Cystatin	0.25	√	0.7 (1)	-
PDE	0.22	√	1.1 (2)	1.4 (1)
NGF	0.20	√	0.8 (1)	-
AChE	0.14	√	0.1 (1)	-
Neprilysin	0.11	√	0.7 (2)	-
Hyaluronidase	0.07	-	-	-
PLB	0.07	√	0.2 (1)	-
Waprin	0.06	√	-	-
DPP IV	0.02	-	-	-
5' Nucleotidase	0.02	-	0.4 (1)	-
CTL	0.01	√	-	-
VEGF	0.01	√	-	2.7 (2)
Total	100		100.0 (45)	100.0 (73)

^a^The relative abundance of a particular transcript was expressed in percentage of total FPKM of all toxins. Values shown are percentages sorted according to toxin gene families

^b^The detection of transcript sorted according to toxin gene family. Exact quantitative values were not provided in the original report. √ indicates detection

^c^The relative abundance of a particular toxin was expressed in percentage of total mean spectral intensities of all toxins (adjusted to gel intensity). Values shown are percentages sorted according to toxin protein families. The numbers of subtype within each family are indicated in brackets

^d^The relative ‘presence’ of a particular toxin was expressed in percentage of the total number of all toxin subtypes (n = 73). Values shown are percentages sorted according to toxin protein families. The numbers of subtype within each family are indicated in brackets

## Discussion

### Venom-gland transcriptome

High redundancy was observed overall in the toxin genes, indicating that multiple toxin isoforms are highly expressed in the venom gland in contrast to non-toxin genes. The phenomenon supports the hypothesis that the toxins were products of repeated gene duplications which underwent molecular adaptation for subsequent neofunctionalization: the evolutionary mechanism proposed to be behind the astonishing molecular diversity of snake venoms [4]. Sixty three of the 128 toxin transcripts show high homology with sequences previously reported for *Ophiophagus hannah*, while the other sequences (> 50 %) are at variable degrees of similarity to toxins from other taxa, consistent with the view that toxin genes expanded from a limited pool of protein families among the advanced snakes [2, 4].

The MOh venom-gland transcriptome reflects the use of venom as an innovated predatory strategy. The role of 3FTxs as the principal toxins in MOh venom is supported by the fact that they are the most abundantly expressed and the most isomerically diverse toxin genes. The 3FTx mRNA expression level in our study appears to be higher (85 %) than that reported by Vonk et al. [20] in a Balinese specimen (about 65 % but unspecified) (Table 1). A total of 18 unique 3FTx transcripts are identified and classified as short-chain (5), long-chain (3) and weak (7) neurotoxin isoforms, as well as neurotoxin-like hypothetical proteins (3) (Fig. 5a). In king cobra envenomation, the neurotoxins are thought to be the major pathogenic cause of rapid death mediated through neuromuscular paralysis and respiratory failure, attributable to the curare-like neurotoxins of the 3FTx family [28, 29]. From the venom-gland trasncriptome, high expression levels were also noted for the genes of SVMPs and PLA_2_s (2-4 % of total toxin transcripts). SVMPs and PLA_2_ are two enzymes commonly present in snake venoms across the Elapidae and Viperidae [21]. The SVMP genes of MOh appear highly diverse (18 transcripts); the multiple isoforms are suggestive of the vast biological activities of the enzyme. The PLA_2_ transcripts, on the other hand, are comprised of only 2 distinct sequences, representing a finding of low sequence diversification similar to that in LAAOs (3 distinct transcripts). The enzymatic products of these genes are not usually involved directly in lethality but play ancillary roles in causing tissue inflammation and destruction (further details are discussed below). Meanwhile, approximately 65 % of the non-toxin genes appear unrelated to envenomation. Most of these are housekeeping genes associated with cellular metabolisms, and they are transcribed at very low FPKM levels, marked with low redundancies.

### Venom proteome

When used in combination, the next generation transcriptomic and proteomic analyses can provide a powerful approach to unveil the molecular complexity of venom toxins. This approach was adopted in the current study for a better correlation of the two processes of gene expression and protein synthesis in the venom glands of the Malaysian king cobra. Among the many proteomic methods, we employed a sequential method of 1D-SDS-PAGE followed by in-gel digestion and nano-ESI-LCMS/MS. A simpler method of whole venom in-solution LCMS/MS is time-saving, cost-friendly, and has been tested out in several venomic studies [20, 30, 31]; however, additional steps of venom protein separation such as SDS-PAGE prior to LCMS/MS are known to be effective to “de-complex” the venom and thus greatly enhance the protein identification by LCMS/MS [32].

In this study, the 1D-SDS-PAGE pattern of MOh venom is similar to the observation reported in Chang et al. [19]. The authors deduced that the prominent bands of king cobra venoms were represented by LAAOs and SVMPs (53-65 kDa), CRISPs (27 kDa), PLA_2_s and 3FTxs (<14 kDa), without reporting their relative abundances. Our approach in examining the protein composition throughout the electrophoretic passage has made it possible to estimate the relative abundance of these toxins, in addition to the detection of several other proteins. While the genomic disclosure by Vonk et al. [20] has been an excellent reference for the evolution and toxin diversity of the Balinese king cobra venom, the individual toxin abundance has not been reported. Our current study that revealed the relative abundance of toxins in MOh venom is thus hoped to provide further insights and correlation between the tissue transcriptome and toxin expression profiles in king cobra. It is however worth noting that toxin variations can still exist between venoms of different geographical origins.

It is interesting to note that 3FTxs and PLA_2_ in MOh venom made up approximately 47 % of the overall abundance, apparently less than in the venoms of local elapids such as *Naja* cobras and kraits, where the total abundance of these two major toxins can be as high as 70-80 % [33, 34]. Instead, a substantial amount of SVMPs (> 20 %), CRISP (> 5 %) and LAAOs (> 5 %) were found expressed in the MOh venom. A hypothesis for the difference could be that the non-neurotoxin components act synergistically in MOh venom in order to subdue neurotoxic elapids such as cobras, which are known to be resistant to neurotoxins [35].

### Comparison of transcriptome and proteome

The expression of mRNAs and protein abundance are not necessarily identical due to various cellular regulatory processes e.g., post-transcriptional, translational and protein degradation that control the steady-state protein abundances [36]. Moreover, the correlation between transcriptomic and proteomic abundances in snake venom has traditionally been poor [37]. It should be noted that in the correlation study, the protein expression is ideally measured based on peptide unit, analogous to the analysis for RNA expression described by Mortazavi et al. [38] in order to correct for the molecule size of messenger transcript and protein. Using this approach, Aird et al. [31] reported a significant correlation of the data (from transcriptome and proteome) for two East Asiatic crotalids, although some variances in the results remained unexplained possibly due to biological as well as analytical factors [31]. Our study on the MOh specimen, however, showed no significant correlation between the venom-gland transciptome and the venom proteome. The correlation may be influenced by many factors, one of which is the time span between venom collection and gland tissue harvesting. In this study, the venom glands had been stimulated by venom milking several days prior to their harvesting as a step to maximise the mRNA synthesis. Admittedly, toxin components can be synthesized at different rates and hence the respective metrics of mRNA and protein expressions taken at one particular time point may not be well correlated. In addition, extensive post-translational modifications occur in many complex toxins such as SVMPs; hence the mature proteins produced may not be necessarily congruent to the mRNA expressed. These factors may explain some of the variances in the data presented here and elsewhere [31, 37]. In the following section, we further discuss the expression of MOh toxins according to their protein families.

### Major transcripts and venom constituents

#### Three-finger toxins (3FTxs)

Three-finger toxins (3FTxs) comprise a family of non-enzymatic polypeptides of 60-74 amino acid residues with four or five disulfide bridges [39]. 3FTxs are usually the principal toxins found in the venoms of elapid snakes such as king cobra, *Naja* cobras, coral snakes, kraits, mambas and sea snakes [21, 40, 41]. The genes may have been recruited before the divergence of different venomous snakes, as low levels of 3FTx transcripts have been reported in the venom-gland transcriptomes of some viperids and colubrids [31, 42–44]. The classification of 3FTxs in general is made based on the number and position of disulfide bonds. Short-chain 3FTxs (S-3FTxs) possess only four disulfide bonds, while long-chain 3FTxs (L-3FTxs) have five with an additional disulfide bond at the distal end of loop II. “Non-conventional 3FTxs” (NC-3FTxs) also have five disulfide bonds, with the fifth bond located within loop I of the molecule [28] (Fig. 5a).

The molecular diversity of 3FTx genes of MOh is well reflected by the presence of multiple unique 3FTx transcripts. The two main 3FTxs of MOh, MOh_FTX01 and MOh_FTX02, share sequence consensus to most curare-like α-neurotoxins as shown in Fig. 5b. MOh_FTX03 is similar to an uncharacterized 3FTxs (S6CB76) in the database with an unknown mechanism of action. L-3FTxs constitute the majority of 3FTx transcripts in the venom gland, notably MOh_FTX01 (74.04 %) (Additional file 2). Interestingly, MOh_FTX01, a long-chain neurotoxin, has an N-terminal peptide sequence (21 terminal amino acids) not seen in other L-3FTxs but identical to that of a weak neurotoxin, WNTX-33 (Fig. 5b). The sequence in the central core of MOh_FTX01 is nevertheless similar to most other long-chain neurotoxins. It is known that the functional sites of long-chain neurotoxins e.g., α-cobrotoxin/elapitoxin from *Naja kaouthia* reside mainly in the second loop of the toxin, with important residues of K23, D27, R33 and K49, while the first loop is not involved in the toxin function [45]. MOh_FTX02 and MOh_FTX03 were found to contain all the critical residues except that the major L-3FTxs, MOh_FTX01 showed substitution of K23 with glutamine and K49 with asparagine. The effect of variations on these critical residues were unknown.

Among the S-3FTx transcripts identified, the three most abundantly expressed isoforms (MOh_FTX06, MOh_FTX07 and MOh_FTX09) are homologous to neurotoxin-like protein 1, hypothetical protein L345_15308 (which was also similar to neurotoxin-like protein NTL2) and muscarinic toxin-like protein 3 homolog, respectively (Fig. 5c). These transcripts belong to the 3FTx “orphan group” (atypical 3FTxs); with the exception of the muscarinic toxin-like protein, the functions of the other two neurotoxin-like proteins are not well understood [46]. A similar type of transcript has also been reported in Balinese *O. hannah* [20], suggesting that some atypical 3FTxs are conserved . Site-directed mutagenesis and nicotinic cholinergic receptor (nAChR) binding studies using the short-chain α-neurotoxin, Erabutoxin a (from the sea krait *Laticauda semifasciata*), revealed that the functional sites of the molecule are distributed over the tips of all three loops in the toxin, including S8 and Q10 in loop I and K27, W29, D31 and R33 that play important roles in short-chain neurotoxin selectivity and affinity to nAChR [47, 48]. In contrast, the functional site of L-3FTxs lies mostly within the second loop of the toxin. The lack of the critical resides Q10 in MOh_FTX06; Q10 and W29 in MOh_FTX07; Q10 and K27 in MOh_FTX09 (Fig. 5c) implies their potential structural-function differences in the MOh venom. Although they are categorized as S-3FTxs (carrying 4 disulfide bonds), the unusual N-terminal amino acid sequences presented in these transcripts may influence the toxin-receptor binding as the tip of the first loop of the typical short-chain neurotoxin, Ea has been shown critical for its binding with the receptor [48]. Three other S-3FTx transcripts encode a typical short-chain neurotoxin SNTX11 (MOh_FTX08), a Type A muscarinic toxin (MOh_FTX010) and a cytotoxin, cardiotoxin-3 (MOh_FTX11), all at extremely low expression levels (0.01-0.03 % of toxin FPKM). Their roles in envenomation have not been elucidated through functional studies.

On phylogenetic analysis, S-3FTx, L-3FTx and NC-3FTx were grouped into separate clusters on the phylogenetic tree (Fig. 6). Genes for both the S-3FTxs identified in MOh venom proteome are basal in the phylogenetic relationship. The finding supports that 3FTx genes of MOh evolved from short-chain isoforms which later adapted and diverged into genes encoding non-conventional 3FTxs and long-chain 3FTxs. The phylogenetic analysis also showed that the main L-3FTx, MOh_FTX01 is more related to the long-chain neurotoxin OH55/56 of the Balinese specimen [20], but distantly diverged from α-cobrotoxin/elapitoxin, indicating their distinctness from the long-chain neurotoxins of *Naja* cobra (Fig. 6).

On the venom proteome, the number of expressed MOh 3FTxs in this study (n = 13) is less than that reported for the Balinese specimen (n = 31, whole-venom shotgun analysis) [20] (Table 1). It is however comparable to the number of 3FTxs reported for the Thai specimen (n = 16, 1DE-shotgun method) [25] and is more diverse than that of the other two Malaysian king cobra samples studied using chromatography separation (n = 7) [19] and 2DE method (n = 7) [24]. The discrepancies could be due to the use of the sensitive nano-LCMS/MS in our study, or geographical variations of the venom within Malaysia (exact locality of the snake is unknown in [19] and [24]). The current study further showed that the L-3FTxs and S-3FTxs account for 62.1 % and 28.8 % of total 3FTx abundance in the proteome, while the non-conventional 3FTxs constitute the least (< 3 % of total 3FTxs) (Additional file 3). In term of all protein abundance, the amount of 3FTxs in MOh venom is hence 43.0 %, a finding comparable to Chang et al. [19] (44.1 % w/w), although the 3FTx subtypes varied between these two studies. While Chang et al. [19] concluded that S-3FTxs constituted 41 % w/w of their Malaysian venom sample with L-3FTxs constituting only 3 % w/w, we demonstrated a lesser extreme distribution of S-3FTx:L-3FTx with a ratio of 1:2 (in relative abundance). The major form of L-3FTx by toxin abundance is nonetheless same in both studies, viz. OH55 (a lethal long neurotoxin, hannalgesin, and a homolog to MOh_FTX01 transcript). Similarly, the 3FTxs in our MOh venom appear to be primarily neurotoxins of variable isoforms, with a very minimum content of cytotoxin or cardiotoxin (0.5 % of total abundance), a finding comparable to Chang et al. [19] (0.4 % cytotoxin). Interestingly, muscarinic toxin-like proteins (S-3FTx subtype) and several NC-3FTxs were expressed in MOh venom at trace amounts; these isoforms have not been reported in the previous studies of Malaysian specimens [19]. The cytotoxin and non-conventional 3FTxs of low abundance in MOh venom likely play minimal role in the lethal action of the venom in view of their minute amount and the higher LD_50_ value (indicating lower toxicity) compared to alpha-neurotoxins [41]. The proteomic presence of long neurotoxin OH56 in the MOh venom also contrasts with the previous conclusion [19] which proposed this to be one of the unique 3FTx markers exclusive for Chinese king cobra (of Guangxi and Hainan origins) and not supposed to be present in the Southeast Asian specimens. Our finding hence indicates that the 3FTx profile of king cobra may be more complex and diverse than previously thought. Nonetheless, the presence of a large number of 3FTx variants in high abundance also implies the possibilities of variable protein structure folding, toxin-receptor interaction and potency. In addition, the immunogenicity of the venom is likely determined by the abundant 3FTxs; the small molecular sizes and diverse epitopes of 3FTxs can be a challenge to the production of a high efficacy antivenom.

Using a computerized model to analyse long NTX sequences retrieved from UniProtKB database, Danpaiboon et al. [25] predicted that a total of 7 amino acids in a king cobra long NTX (Q2VBP4, Chinese origin) interacted with the human single-chain variable antibody fragments specific to *N. kaouthia* long NTX (NkLN-HuScFv) at the following 7 amino acids: C26, W29, G31, R33, K36 in loop 2, and R47, N49 in loop 3, hence supporting the apparently weak cross-neutralization effect of NkLN-HuScFv against the lethal effect of king cobra (Thailand) in mice. However, matching of the said interactive amino acids of Q2VBP4 to the major NTXs (abundance > 10 %) in MOh venom (OH55, OH56, LNTX2, MOH_FTX07 and neurotoxin-like protein 1) yielded limited similarity (10-40 % matching). This suggests that NkLN-HuScFv may not be effective in neutralising the king cobra venom from Malaysia. From the practical standpoint, Leong et al. [49] confirmed in a mouse model that Thai *Naja kaouthia* monovalent antivenom (NKMAV) has low neutralization capacity against MOh venom, indicating the antigenic dissimilarities of both venoms despite the conserved protein scaffolds between neurotoxins of MOh and *N. kaouthia*. This is not surprising considering the fact that the antivenom manufacturer has to combine venom immunogens from both king cobra and *N. kaouthia* (in addition to two *Bungarus* species) in order to produce the effective Neuro Polyvalent Antivenom (NPAV). Clinically, an attempt to treat Malaysian *O. hannah* envenomation in Kuantan, Malaysia with NKMAV has proven in vain as the antivenom against *N. kaouthia* failed to cross-neutralize both the systemic and local effects from the bite [50]. In the MOh envenoming case, the systemic effects were only arrested much later with the arrival and administration of the king cobra-specific antivenom.

We postulate that the neurotoxins of MOh are highly distinct to those of *Naja* cobras’ as a result of adaptive evolution and positive selection to ensure that the MOh neurotoxins are sufficiently effective to bind *Naja*’s nicotinic receptor since *Naja* cobra is a prey top in king cobra’s food list – a known predator–prey phenomenon in palm oil estates in Malaysia. Generally, *Naja* cobra is resistant to NTX as their receptors have been mutated with a sugar chain that masks the binding by NTX [35]. In addition to distinct neurotoxins, the MOh venom proteome also reveals abundant high molecular mass proteins (mainly enzymes) not commonly present in large amount in *Naja* cobra venoms, implying their potential roles in killing and digestion of other neurotoxic snakes by king cobra. The higher share of non-3FTx components (e.g., CRISP, SVMPs, LAAOs, vespryn; generally have lower lethality) in the MOh venom may be a reason for the venom’s higher LD_50_ (> 1 μg/g, *i.v.*) in mice compared to that of *Naja* venom (for instance, 0.2 μg/g for Thai *N. kaouthia*) that is predominated with alpha-neurotoxins [40, 49]. This is supported by an earlier report [51] where the major neurotoxic fraction of MOh venom, purified by ion-exchange chromatography and accounted for ~23 % venom weight, has an *i.v.* LD_50_ of 0.23 μg/g mice, a value disproportionally lower than that of the crude venom (> 1 μg/g).

#### Snake venom metalloproteinases (SVMPs)

From the MOh venom-gland transcriptome, the SVMP transcripts encode at least 15 unique SVMPs, with sequence similarities matched to SVMP-ohanin, SVMP-cobrin, SVMP-BmMP, SVMP-kaouthiagin, SVMP-mocarhagin-1, SVMP-atrase-A, Chain-A-SVMP, asrin and carinatease-1 from different elapid snakes (Fig. 7; Additional file 4). The sequence divergence is evident, supporting the view of extensive diversification of SVMP functions under accelerated evolution [11]. All the identified SVMP transcripts belong to the P-III class (with metalloproteinase/ disintegrin/ cysteine-rich (MDC) domains), the SVMP type that has a rich array of biological activities, including potent haemorrhagic activity, inflammation, apoptosis, prothrombin activation, and inhibition of platelet aggregation [52]. From the SVMP transcript sequences of MOh, we identified the absence of the seventh cysteine residue in Domain M that takes part in the disulfide bond exchange for autolysis during secretion or formation of the biologically active DC domain, typical for Viperidae SVMP-PIIIs [52, 53]. Figure 7 shows that the MOh and annotated SVMP-PIII (except asrin A6XJS7) M domains from several elapids have only six cysteine residues, consistent with previous studies [52] including that for Balinese king cobra [20]. The effect of structural difference as such has been shown by Guan et al. [54] that no autolytic activity was observed during the preparations of two P-III SVMPs sourced from the Taiwanese *Naja atra*. Thus, the SVMP-PIIIs of Malaysian *O. hannah* and most elapids likely exhibit the similar structure-function properties distinct from those of viperids.

Some proteomic studies indicated that SVMPs are expressed mainly in the Viperidae and Colubridae venoms, and in a relatively smaller amount in Elapidae venoms [53]. Our findings revealed that SVMPs are present in a substantial amount in the MOh venom, with a total of 12 proteins detected. Our finding of diverse and abundant SVMPs in MOh venom is in agreement with Vonk et al. [20] on the view that SVMPs are important pathogenic toxins besides 3FTXs in the Balinese king cobra venom. This is also supported by Chang et al. [19] who reported high metalloproteolytic enzyme activity in king cobra venoms, although the authors did not determine the subtypes and composition of SVMPs in the venoms. On the other hand, SVMP was not identified at all in the previous 2DE proteomic study of Malaysian king cobra [24], a rather peculiar finding that raises a conflict in local venom database, possibly due to inadequacy of the methodology. Earlier studies indicated that at least five SVMP isoforms with proinflammatory and oedema-inducing activities were present in king cobra venoms, two of which were proven to be hemorrhagic proteases [55]. The major hemorrhagic protease, termed hannahtoxin, is a 66-kDa zinc-dependent metalloprotease that exhibited species-specific lethality, where it is highly lethal to rabbit but not to mice at comparable doses [56, 57]. Although local hemorrhage is not a well-recognized clinical feature of king cobra envenoming, the proteolytic and proinflammatory activities of SVMP may be involved in cytotoxicity leading to necrosis and tissue destruction.

#### Cysteine-rich secretory proteins (CRISPs)

Multiple sequence alignment [Fig. 8] of the 7 CRISP transcripts shows that the sequences are highly conserved across different snake species and the complete sequence of MOh_CRP03 obtained is almost identical to ophanin sequence derived from UniprotKB database, including its functional region known for the inhibition of smooth muscle contraction [58]. This is in agreement with the transcriptomic finding reported for the Balinese king cobra, where CRISP is found with limited diversity and little evidence of gene duplication [20]. This gene family likely has conserved functional activities across most lineages, and does not participate in the evolutionary arms race seen in the more pathogenic toxin families such as 3FTx.

The presence of CRISPs in king cobra venom proteome varies between studies. CRISP is not reported in two previous studies on the venom proteome of Malaysian king cobra [19, 24]. In the present study on MOh venom, one CRISP isoform was identified, consistent with the finding from the Thai specimen [25] but varied from the Balinese which recorded a total of 5 CRISP isoforms [20]. In MOh venom, opharin precusor is the identified CRISP isoform. This toxin has the putative function of reducing smooth muscle contraction that is elicited by high potassium-induced depolarization. The degree of inhibition by opharin is however lower than most other CRISPs [58], possibly due to the critical amino acid difference essential for channel activity inhibition: as with ophanin, the translated MOh CRISP possesses Y189 in substitution of F189 (present in strong contractors) and Y186 instead of E186 (present in most blockers of smooth muscle contraction) (Fig. 8). Although the pharmacological activity of ophanin as a weak antagonist of smooth muscle contraction has been characterized, its exact role in envenomation remains elusive especially in MOh which venom contains a considerable amount of this toxin.

#### L-amino acid oxidases (LAAOs)

The LAAO transcripts revealed by the MOh venom-gland transcriptome consist of the three main functional domains [59] and share high sequence similarity with sequences of other king cobras (retrieved from the UniprotKB database) (Fig. 9). The major transcript sequence (MOh_LAO01) in our study is highly homologous to the sequence of hypothetical protein L345_17374 from the Balinese king cobra [20] and king cobra OH-LAAO (P81383). Unlike 3FTxs (that have values of transcript redundancy 10 times higher than LAAOs), LAAOs appear rather well conserved within king cobras from different regions.

Snake venom LAAOs are reported present natively as a homodimer with molecular mass around 120 kDa, while under reducing conditions, present as subunit of around 50-70 kDa [60]. We showed in our proteomic study that the LAAOs of MOh venom distributed mainly in gel section 3 corresponding to 55-75 kDa (Fig. 3). Despite the relatively lower mRNA expression, the protein comprises close to 6 % of MOh venom (Additional file 3), comparable to the approximation of 8 % w/w in an earlier study of Malaysian king cobra LAAO [10]. The peptide sequences exhibit low variability with only two isoforms identified in the proteome, comparable with the Thai and Balinese samples where one LAAO subtype was identified [20, 25]. Our finding is also in agreement with Chang et al. [19] who reported substantial LAAO activity in four king cobra venoms of different localities, although the paper reported neither the compositional subtype nor the abundance of this enzyme. These studies however strongly disagree with another proteomic study on a Malaysian king cobra sample where no LAAO was detected at all [24].

The substantial amount of LAAO in the MOh venom proteome (> 5 %) suggests that the toxin may serve beyond ancillary function. Its role in king cobra predation and envenomation may be related to its potent proinflammatory and cytotoxic activity [60] that can induce extensive tissue destruction. In addition, the functional uniqueness of king cobra LAAO has been shown on its substrate specificity compared to other snakes, where its heat-stable property contributes greatly to its potential for biomedical application [10]. Notably, LAAO from the Malaysian king cobra has been shown to exhibit potent *in vitro* and *in vivo* selective cytotoxicity [60, 61]. Further characterizations of king cobra LAAO is therefore a feasible and promising research direction towards cytotoxic drug discovery.

#### Vespryn (Ohanin)

The complete sequence of the only vespryn transcript in MOh matches identically to the ohanin sequence (Fig. 10). The sequence, covering signal and mature chain, is also identical to the corresponding sequence regions reported for the Balinese specimen [20] (hypothetical protein L345_13461). The Balinese king cobra vespryn sequence, however, contains additional pre-signal 30 amino acid residues (Fig. 10), the significance of which is uncertain. In contrast to other members of snake venom proteins, ohanin presents a unique single cysteine residue (residue 52), and the protein sequence shares little similarity to the PRY-SPRY domains (B30.2-like domain) with relatively short N-terminal extension [62]. Although Thaicobrin (P82885) isolated from the monocled cobra, *Naja kaouthia* was deposited in the protein database much earlier than ohanin, there is no published literature and little characterization work has been done. Together with Thaicobrin, the distinct sequence of ohanin has made them the first member of vespryns (venom PRY-SPRY domain-containing proteins) family sourced from snake venom.

Pung et al. [16] reported the presence of ohanin at a low abundance (~1 mg/g) in king cobra venom sourced from Jakarta, Indonesia. In contrast, the MOh venom proteomic study shows the venom contains a higher amount of ohanin (5.7 %, ~57 mg/g) (Additional file 3) that were distributed mainly in gel sections 10 and 11 (Fig. 3), corresponding to the known molecular mass of ohanin (~12 kDa). The difference in ohanin content in *O. hannah* venoms from Malaysia (MOh) and Indonesia (Jakarta) may seem to represent a remarkable geographical variation of the venoms.

#### Phospholipases A_2_ (PLA_2_s)

The two complete PLA_2_ transcripts (MOh_PLA01 and MOh_PLA02) of MOh were annotated to Group IA (PLA_2_-2) and IB (PLA_2_-1), respectively, similar to the PLA_2_ enzymes in Chinese *O. hannah* venoms [15, 63], and they share the characteristic Asn^6^ N-terminus for venom PLA_2_ of king cobra (Fig. 11). The major PLA_2_ (4 % of abundance) expressed in the proteome of MOh venom is the acidic PLA_2_-2 (Group IA subtypes), consistent with Tan and Saifuddin [14] who reported that the major PLA_2_ of Malaysian king cobra was an acidic isoform constituting 4 % of the venom weight. The enzyme was however non-lethal in mice at an intravenous dose of 10 μg/g but exhibited moderate anticoagulant and oedema-inducing (proinflammatory) activities, in contrast to the Fujian PLA_2_-1 (Group IB subtype) that was mildly lethal and cardiotoxic in mice [63]. Remarkable variations are noted within the Group I PLA_2_ among king cobra venoms of different origin: while we identified Group IA PLA_2_ from MOh, Huang et al. [63] isolated only Group IB PLA_2_ from a Fujian Chinese sample; Vonk et al. [20] identified two each for Group IA and Group IB from a Balinese sample; Vejayan et al. [24] identified one each of Group IA and Group IB from a Malaysian sample; Danpaiboon et al. [25] identified 2 Group IA and 1 Group IB PLA_2_ from a Thai sample. With the inconsistency of king cobra PLA_2_ subtypes identified, the comparative analysis is further compounded by undetermined toxin abundance in each study. Chang et al. [19] showed with quantitation in another Malaysian sample the presence of 2 Group IA and 1 Group IB PLA_2_ subtypes, however each with exceptionally low abundance ranging from 0.16-0.32 % only, indicating that the total PLA_2_ content in the venom was far less than 1 % [19]. The intraspecific PLA_2_ variations are therefore vast and may be related to not only geographical distribution but also ontogenic factors. The current proteomic study, nonetheless, shows that the MOh major PLA_2_ is the acidic, proinflammatory but non-lethal variant of Group IA PLA_2_. It constitutes a small portion of the Malaysian king cobra (MOh) venom, approximately 10 times less than PLA_2_s found in the regional *Naja* cobra venoms (approx. 20-30 %) [34, 40]. In view of the negligible cytotoxin content and small amount of PLA_2_, local tissue necrosis that occurs in MOh envenoming is possibly induced by other cytotoxic components in the venom, for instances, SVMPs and LAAOs that exist at a high abundance.

#### Cobra venom factors (CVFs)

The trasncripts of cobra venom factors from the MOh transcriptome are highly homologous to OVF, Ophiophagus venom factor, the CVF first purified from the venom of a Chinese (Guangxi) king cobra [64]. Interestingly, the OVF transcripts in MOh transcriptome reveal 9 amino acid residues (545-553) absent in the reported sequence for Guangxi OVF, but present identically in that of the Balinese specimen [20]. This suggests that the structures of OVF from king cobras of the two Southeast Asian regions share high similarity and are sufficiently distinct from that of the China specimen. In addition, two novel regions (residue 3-42 and 504-544) not reported previously in CVF/OVF have been identified in the OVF transcript of MOh (Fig. 12). BLAST analysis of these peptides retrieved no identifiable protein or peptide region. The role of the additional amino acid sequences in the structure-function relationship of Malaysian CVF/OVF remains to be investigated.

The number of CVF subtypes expressed in the MOh venom (n = 4) is comparable to that reported in the Balinese specimen (n = 5) [20], but more diverse than the Thai specimen and another Malaysian sample [24, 25]. CVF is a protein with high molecular mass (approx. 150 kDa) and it dissociates into α, β and γ chains under reducing condition [64], consistent with our findings where the gel sections 3, 5, 6 and 7 (Fig. 3) corresponded to the reported sizes of α (72kDa), β (45kDa) and γ chain (32kDa). CVF is not directly lethal; it facilitates the absorption and spread of venom from the bite site, as CVF-activated local complement system releases anaphylatoxins C3a and C5a that increase the regional vascular permeability and blood flow [65].

### Low-abundance transcripts and toxins

#### Toxins constituents expressed in both venom-gland transcriptome and venom proteome

Kunitz/BPTI serine protease inhibitors (KUNs) transcripts consitutute 1.84 % of toxin FPKM, while one KUN was expressed into the venom proteome at 1.0 % of toxin abundance. The expressed KUN protein is similar to OH-TCI, the first reported KUN from snake venom that exhibited equivalent trypsin and chymotrypsin inhibitory activities [17]. In addition, cystatin, an inhibitor of various C1 cysteine proteases that may play a role in preventing toxin auto-digestion [66], was detected at a low amount in the transcriptome (0.25 % toxin FPKM) and proteome (0.65 % toxin abundance). In comparison, the Balinese and Thai king cobra venoms contain, respectively, 3 and 1 KUN subtypes but cystatin has not been reported [20, 25]. The exact function and action of KUNs remain to be elucidated.

From the MOh venom-gland transcriptome, we have identified 3 transcripts (0.11 % total toxin FPKM) that encoded neprilysin, a metalloendopeptidase with affinity for a vast range of physiological targets, including natriuretic and vasodilatory neuropeptides for their regulation [67]. Novel metallopeptidases exhibiting similarity to neprilysin was first demonstrated in the venom-gland transcriptomic study of *Echis* sp. [68], and recently the neprilysin transcript was revealed too from the venom gland of the Balinese king cobra, but its expression in the king cobra venom proteome was not reported [20]. In this study, we showed that neprilysin is present in the venom proteome of Malaysian king cobra, although its identity and function as a secretory toxin in the venom are still elusive at this stage of venom study.

From the venom gland transcriptome, three transcripts were identified for encoding snake venom serine proteases (SVSPs) at rather low FPKM (0.17 % abundance of total toxin transcripts). Through LCMS/MS and homology search, the expressed peptides were noted similar to alpha- and beta-fibrinogenase OhS1 (0.7 % of venom protein abundance). In comparison, 3 SVSPs and 1 kallikrein were identified from the venom proteome of Balinese and Thai specimens, respectively [20, 25] but not reported in the other two Malaysian samples [19, 24]. The serine fibrinogenase in MOh venom has a putative role of a potent fibrinogenolytic and amidolytic agent without haemorrhagic effect [69]. Serine proteases at 5-7 % in viperid venom is able to elicit significant coagulopathic effect *in vivo* and *in vitro* [70]; this effect however has not been a well-recognized clinical syndrome in king cobra envenomation, and laboratory test (APTT) using human plasma for the venom’s procoagulant effect has not been positive [19]. In addition to the fibrinogenolytic toxins, phospholipase B (PLB) with haemolytic activity from *Drysdalia coronoides* [71] was detected in a minute amount in the MOh transcriptome and the venom proteome. This enzyme was also identified in the venom proteome of the Thai specimen [25] but not reported from the other sources [19, 20, 24].

Acetylcholinesterases (AChEs) are usually expressed at low level in snake venoms; the highest abundance ever reported was 0.8 % (w/w) from the genus *Bungarus* (krait) [72]. The major isoform of AChE transcript from MOh exhibits extremely high homology to AChE full sequence of *Bungarus fasciatus* (Q92035), supporting the antigenic cross-reactivity observed between king cobra AChE and the monoclonal antibodies of krait AChE [72]. One AChE (MOh_ACE01) was detected in the MOh venom proteome at a relatively low abundance (0.1 %). The presence of this enzyme in king cobra venom was previously identified only through functional study. Chang et al. [19] showed that the AChE activities of Malaysian and Thai king cobra venoms were several-fold higher than the Indonesian and Chinese samples, and described an inverse relationship between the AChE activity and the neurotoxic fatality of the venom. However, the postulation of the ‘inverse relationship’ seems contradictory as it was suggested that the immediate acetylcholine hydrolysis at synapses might reduce the competition between the neurotransmitter (acethylcholine) and the curare-like neurotoxins, thus potentiating the neurotoxicity. Besides, although venom AChEs may interrupt musculatory control through rapid degradation of ACh, their effect on cholinergic transmission in the central nervous system and autonomic ganglia remains unclear [73]. Systemic hypotension may be a possible effect associated with the interruption of cholinergic transmission at the autonomic ganglia, thus favouring prey immobilization.

The transcription of nerve growth factor (NGF) genes in the MOh venom glands is consistent with the findings for the Balinese king cobra [20]. A small amount (0.8 %) of NGF was also detected in the MOh venom proteome. Of note, the proteomic presence of this minor toxin in king cobra venoms has not been reported nor quantitated previously [19, 20, 24, 25]. It has been suggested that NGF can exhibit arginine esterase activity that contributes to hypotensive effect through nitric oxide liberation and histamine release [31]. Interestingly, this is a group of snake venom constituents, after the widely-studied anticoagulant, antiproliferative and antimicrobial toxins, shown to have therapeutic potential for neurodegenerative brain disorders, e.g., Alzheimer’s disease [74, 75].

Phosphodiesterase (PDE) and 5’-nucleotidase were two other minor toxins detected in both the venom-gland trasncriptome and venom proteome of MOh. Both the enzymes were detected in the venom proteome of Thai king cobra [25], while only PDE was identified from the Balinese specimen [20]. These enzymes primarily act to liberate nucleosides which may help in prey immobilization [76–78]. Venom phosphodiesterases are known to cause locomotor depression and reduction of mean arterial blood pressure [79]. Together with 5’NUCs and toxins with hypotensive and immobilising potentials such as OVF, AChE and NGF, these components may act synergistically to enhance prey subduing.

In comparison with the study on Balinese king cobra [20], we noticed some variations in the “–omic” findings between king cobras from the two geographical regions (Table 1). Besides the variabilities of sequence and abundance described, we also found the inconsistent presence of a number of toxins between the two specimens. For instance, NGF, AChE, neprilysin, PLB, cystatin and 5’-nucleotidase present in the MOh proteome were not reported in the Balinese specimen proteome [20]. Conversely, natriuretic peptide (NP) and vascular endothelial growth factor (VEGF) were detected in the venom proteome of the Balinese specimen [20] but absent in the MOh although the expression was detected at low mRNA level. One interesting, consistent feature shared by both king cobras from the Malayan Peninsula and Bali Island is the absence of lectin in the venom proteome in spite of their gene expression in the venom glands. This observation supports that lectins do not contribute to king cobra envenoming [20] which is in contrast to many other venomous snakes particularly from the Viperidae family [20, 70]. In addition, although the two specimens did not differ remarkably in the composition of major pathogenic toxin components e.g., 3FTxs and SVMPs, variations are noted in the gene sequences (Additional file 4) and possibly proteomic abundances (not reported for the Balinese specimen), indicating intra-specific venom diversity that may lead to variations in immunological profile and antivenom effectiveness.

### Toxins not translated from venom-gland transcriptome

Twenty seven toxin transcripts were identified only at the transcriptional level of MOh venom glands. These transcripts encoded protein genes for insulin growth factors (IGFs), natriuretic peptides (NPs), hyaluronidase, dipeptidylpeptidase IV (DDP-IV), vascular endothelial growth factors (VEGF), waprins and C-type lectins (CTLs), totalled to 1.18 % of all toxin FPKM. The activities of these toxins have not been previously investigated in king cobra venom. It is an assumption that their occurence in the venom is extremely low or negligible since the peptides of these proteins were not detected by the high resolution tandem mass spectrometry.

Transcripts of IGFs have been previously categorized into secretory toxin transcripts by Vonk et al. [20], although its toxic role is uncertain to date. At the proteomic level, this toxin was also not reported in both MOh and the Balinese king cobra venoms, but its presence was detected along with a hepatocyte growth factor in the Thai specimen (abundance was not determined) [25]. Natriuretic peptide, a hormone that controls over natriuresis, diuresis, blood pressure, homeostasis and inhibition of aldosterone secretion in all vertebrates was not identified in the proteomes of both MOh and Thai [25] specimen, but reported in the Balinese specimen proteome [20]. This is another physiological protein which gene was likely recruited earlier into the toxin arsenal; the expression however has been low among venomous snakes. Besides, VEGFs, potential vasodilators, were expressed in the venom proteome of the Balinese specimen [20] but never reported in MOh and the other king cobra venoms studied [25].

Hyaluronidase, DPP-IV, waprin and CTL were consistently not reported in the venom proteomes of king cobras (including the Malaysian and Baliense specimens) although the genes were transcribed in the venom glands [19, 25]. Interestingly, the enzymatic activity of hyaluronidase has been reported previously in king cobra venom [51] but at a level much lower than that of *Naja* sp., *Bungarus* sp. and viperid venoms. In this study, a full sequence of hyaluronidase from MOh venom gland transcriptome was identified. Its high homology to sequences of viperid snakes suggests that the gene and protein function appear to be conserved across many venomous lineages despite its trivial expression in the venom. DPP-IV, waprin and CTL have not been reported thus far in king cobra venom proteomes; but it remains interesting for further study as complete sequences of these toxins have been made available from the MOh venom-gland transcriptome [Additional file 2]. The expression of these genes without translation into proteins indicate their early recruitment into the venom-gland transcriptome, however, adaptation and selection have favored the expression of other toxins to suit the predatory arm-race and ecological niche changes for the king cobras. An alternative possible explanation for this is the convergence of some genes which begin to emerge as potential contributor of toxins in adaptation to the changing niche. Data of these sequences may serve useful purposes for biodiversity study and drug discovery in the future.

## Conclusion

This study demonstrates complex and diverse composition of Malaysian king cobra venom at both the transcriptomic and proteomic levels. Unlike venoms of Southeast Asian *Naja *sp. that mainly comprise neurotoxins, PLA_2_s and cytotoxins, MOh venom contains a higher degree of toxin complexity predominated with neurotoxins and SVMPs, in addition to appreciable amounts of CRISP, LAAO, CVFs, vespryn, PLA_2_ and NGF. Additionally, the MOh venom contains small amounts of neprilysins and AChE which may have synergistic action on the neurotoxicity of the venom. The findings also suggest the presence of toxins that may induce hypolocomotive and hypotensive effects in the venom, although clinically humans are susceptible more rapidly to neuromuscular paralysis due to the faster absorption of neurotoxins and their intrinsic toxicity with a rapid onset [80]. The unique toxin composition of king cobra venom may serve to aid this ophiophagic species in preying on other snakes especially *Naja* cobras which are known to be neurotoxin-resistant [35].

As anticipated, intra-species variation of venom is not uncommon, and this is exemplified by the king cobras among the peninsular Malaysian (MOh), the Thai and the insular Balinese specimens. We propose the need to address the medical concerns of such phenomenon, where comparative studies in the future should aim to provide variation details in terms of the sequence diversification and the relative abundance of toxins between venoms of different geographical sources. The integrated transcriptomic-proteomic knowledge as such is fundamental for deeper insights into the mechanisms of snake venom toxicity and the natural history of venom evolution. The knowledge may provide crucial information for optimizing the production of an effective pan-regional antivenom for related species in the future.

## Methods

### Snake venom gland preparation

The specimen was an adult *Ophiophagus hannah* measuring 190 cm in total length captured in a suburban vicinity of Seremban (central Peninsular Malaya), and identified by the authors CHT and NHT. Venom was milked from the live specimen by inducing the snake to bite through a clean plastic film stretched over a strile container, while gentle massage was applied to both glands for 20 sec to ensure maximum venom collection. The venom was lyophilized and stored at -20 °C until used. Venom milking was carried out to stimulate the venom gland transcription, while the snake was allowed to rest for four days for the transcription process to be maximized [81]. Following euthanasia, the venom glands were swiftly removed and sectioned into dimensions of < 5x5 mm before preserving them in RNAlater solution at a 1:10 volume ratio. The solution was allowed to permeate the tissues at 4 °C overnight before transferring to -80 °C for storage until further use. The use of snake has been granted by the Department of Wildlife and National Parks, Peninsular Malaysia (#JPHL&TN (lP): 80-4/2), and the procedure was approved as of the protocol for snake venom milking and tissue use for research (#2013-11-12/PHAR/R/TCH) by the Institutional Animal Use and Care Committee (IACUC) of the University of Malaya, Malaysia.

### Total RNA extraction and mRNA purification

The dissected venom gland tissue was submerged and homogenized in a 1 mL glass homogeniser with TRIzol solution (Invitrogen) under sterile condition. This was followed by the addition of 20 % chloroform, centrifugation and RNA-free DNAase I treatment to separate RNA from cellular debris and residual DNA. The isolated RNA was then pelleted with isopropyl alcohol and washed with 75 % ethanol. Polyadenylated mRNA (poly(A)^+^ RNA) was subsequently purified from 20 mg of total RNA using oligo(dT) magnetic beads as per the Illumina manufacturer’s instructions. Two rounds of poly-A^+^ mRNA isolation were performed.

### DNA library construction and sequencing

Enriched poly(A)^+^ mRNA isolated from the total venom-gland RNA was used for cDNA construction. The mRNA isolated was fragmented in standard buffer containing divalent cations (Zn^2+^) into short fragments, which acted as templates for cDNA synthesis. Random hexamer-primer (*N6*) was used to synthesize the first-strand cDNA, followed by second-strand cDNA synthesis with the double-stranded cDNA as input material, using second strand buffer, dNTPs, RNase H and DNA polymerase I. From these cDNA, a paired-end library was synthesized using the Genomic Sample Prep kit (Illumina), according to the manufacturer’s instructions. Short fragments were purified with QIAquick PCR extraction kit (Qiagen) and resolved with *EB* buffer for end repair and the addition of poly(A) by the use of Klenow fragment to aid in the subsequent ligation of the Illumina adaptors, which contain a single thymine (T) base overhang at their 3’ ends. Following the ligation with sequencing adaptors, these short fragments of cDNA were PCR-amplified and electrophorezed on a 1.5 % to 2 % TAE agarose gel. From the electrophoretic agarose gel, suitable fragments (200nt-700nt) were selected as templates for subsequent PCR amplification. Sequencing of the PCR-amplified library was then accomplished in a single lane on the Illumina HiSeq™ 2000 platform with 100-base-pair, paired-end reads.

### Raw sequence data and filtering

Sequenced data from Illumina HiSeq™ 2000 were transformed by base calling into sequence data, called the raw data or raw reads and were stored in fastq format. Raw reads were filtered by removing (i) adaptors; (ii) reads with > 5 % of unknown nucleotides, or (iii) low quality reads with > 20 % of low quality bases (determined as base quality ≤ 10), hence yielding clean data or clean reads.

### De novo transcriptome assembly

*De novo* ‘shot-gun’ transcriptome assembly was carried out with the short reads assembling programme, Trinity [26]. Three independent software modules i.e., Inchworm, Chrysalis, and Butterfly, comprised the Trinity programme and were sequentially applied to process the large volumes of RNA-seq reads. In brief, this was based on the algorithm of *de Bruijn* graph construction which began by aligning k-mers (k = 25), and reads with certain length of overlap were joined to form linear contigs. The reads were mapped back onto contigs, and by referring to paired-end reads, contigs from the same transcript as well as the distances between them were determined. The contigs were then partitioned into clusters, each of which carried a complete set of *de Bruijn* graphs (representing the transcriptional complexity at a given gene or locus). The graphs were independently processed to obtain full-length transcripts for alternatively spliced isoforms, and to tease apart transcripts that corresponded to paralogous genes.

### Transcript clustering

The transcript sequences generated through Trinity were called Unigenes. Unigenes from the transcriptome assembly were further processed for sequence splicing and redundancy removing with TGI Clustering tools (TGICL) to acquire non-redundant transcripts at the longest possible length. The transcripts were then subjected to family clustering, which resulted in two classes of transcripts: (a) Clusters, with a prefix CL and the cluster id behind; (b) Singletons, which id was simply left with a prefix of Unigene. In each Cluster, there are several transcripts which sequence similarities among them being > 70 %; while the singletons ‘Unigenes’ lack overlapping with other fragments at the given stringency.

In the following step, the transcript Unigenes were aligned by BLASTX to protein databases in the priority order of NCBI non-redundant (NR), Swiss-Prot, KEGG and COG, with a cut-off *E* value < 10^−5^. Proteins with the highest ranks in the BLASTX results were referred to determine the coding region sequences of the Unigenes, followed by translation into amino acid sequences (using the standard codon table). Hence, both the nucleotide sequences (5' - 3') and amino sequences of the Unigene-coding regions were acquired. Transcript Unigenes unaligned to any of the protein databases were analyzed by a software named ESTScan [82] to determine the nucleotide sequence (5’-3’) direction and amino sequence of the predicted coding region. The length of sequences assembled was a criterion for assembly success. The distributions of the lengths of contigs, scaffolds, and Unigenes were calculated.

### Functional annotation of the transcripts

The process retrieved proteins with the highest sequence similarity with the given transcript along with their protein functional annotations, recorded in the Annotation Folder. Annotation of the transcripts provides information of the mRNA expressions (*see below*) and the putative protein functions. For functional annotation, the generated transcript sequences were firstly aligned by BLASTX to certain protein databases e.g., NR, Swiss-Prot, KEGG and COG (each with a cut-off E-value < 10^−5^), and aligned by BLASTN to nucleotide databases NT (cut-off E-value < 10^−5^).

### Expression annotation of the transcripts

To determine the transcript abundances for the identified genes, the FPKM method (Fragments Per Kilobase of exon model per Million mapped reads) [38] was used. The formula is shown below:

$$ \mathrm{FPKM}\kern0.5em \mathrm{of}\kern0.5em \mathrm{gene}\kern0.5em \mathrm{A}\kern0.5em =\kern0.5em \frac{1{0}^6\kern1em C}{N\kern1em L/1{0}^3} $$

where FPKM is set to be the expression of gene A, C to the number of fragments (i.e., reads) that uniquely aligned to gene A, N to be the total number of fragments (i.e., reads) that uniquely aligned to all genes, and L to be the base number in the CDS of gene A. The FPKM method is able to eliminate the influence of different gene length and sequencing discrepancy on the calculation of gene expression.

### Venom-gland transcript classification based on toxinology

Data in the Annotation folder (from BLAST analyses) were further studied to determine which transcripts (Unigenes) could be identified as toxin, or non-toxin categories. Keywords were used in search-and-find of the subject description for each toxin match. In view that the final translated toxin products are proteins in nature, the encoded amino acid sequences of each transcript sharing a same keyword description were manually checked for their sequence similarity using Mutalin® software [83], and subjected to BLASTp (protein suite) search to ascertain homology with known toxins. Transcripts for cellular proteins and house-keeping genes were categorized into “non-toxins” while those without significant hits/matches were classified as ‘unidentified’. The relative expression of BLAST-annotated venom-gland transcriptomic Unigenes (in % of the three categories), the relative abundance and the diversity of various toxins in % of (i) total protein-encoding transcripts, and (ii) total toxin-encoding transcripts were determined.

### Redundancy of gene families

In addition, the redundancy of gene was assessed by dividing the transcriptional activity level or transcript reads (FPKM) with the total number of transcripts within a cluster or a group of genes. High redundancy indicates high expression level of a gene group.

### 1D SDS PAGE and in-gel trypsin digestion

SDS-polyacrylamide gel electrophoresis (SDS-PAGE) was conducted according to the method of Laemmli [84] calibrated with the Thermo Scientific PageRuler Prestained Protein Ladder (10 - 170 kDa). The Malaysian *O. hannah* (MOh) venom (100 μg) was loaded onto a 15 % gel and the electrophoresis was performed under reducing condition at 80 V for 2.5 h. Proteins were stained with Coomassie Brilliant Blue R-250 and a total of 12 gel sections were cut across the sample lane. Each gel section was further excised into smaller pieces (~1 × 2 mm) and stored in a 600 μL micro-centrifuge tube. In-gel digestion was performed using in-gel tryptic digestion kit from Thermo Scientific. The gel pieces were destained for 30 min at 37 °C with shaking, and the in-gel proteins were then reduced at 60 °C for 10 min with reducing buffer and alkylated in the dark at room temperature for 1 h. Washing was repeated with destaining buffer at 37 °C for 15 min followed by dehydration using 50 % acetonitrile for 15 min. Gel pieces were allowed to air-dry for 5-10 min, and subsequently incubated with activated trypsin solution at room temperature for 15 min. Digestion buffer were added to the tube and incubate sample at 30 °C overnight with shaking. The digested mixtures were then separated, and further extraction of peptides was archieved by incubated 1 % trifluoroacetic acid solution with the gel pieces for 5 min. The digested protein samples were desalted and concentrated with a C18 ZipTip (Millipore).

### Nano ESI-liquid chromatography and tandem mass spectrometry (nano-ESI-LCMS/MS)

The digested peptide eluates were subjected to nano-electrospray ionization (ESI) MS/MS experiments using an Agilent 1200 HPLC-Chip/MS Interface, coupled with Agilent 6520 Accurate-Mass Q-TOF LC/MS system. Samples were loaded in a large capacity chip 300 Å, C18, 160 nL enrichment column and 75 μm × 150 mm analytical column (Agilent part N° G4240-62010) with a flow rate of 4 μl/min from a capillary pump and 0.3 μl/min from a Nano pump of Agilent 1200 series. Injection volume was adjusted to 1 μl per sample and the mobile phases were 0.1 % formic acid in water (A) and 90 % acetonitrile in water with 0.1 % formic acid (B). A gradient of 47 min (3–50 % solution B for 30 min, 50–95 % solution B for 2 min, and 95 % solution B for 5 min) was applied using Agilent 1200 series nano-flow LC pump. Ion polarity was set to positive ionization mode. Drying gas flow rate was 5 L/min and drying gas temperature was 325 °C. Fragmentor voltage was 175 V and the capillary voltage was set to 1995 V. Spectra were acquired in a MS/MS mode with a MS scan range of 110–3000 m/z and MS/MS scan range of 50–3000 m/z. Precursor charge selection was set as doubly, triply or up to triply charged state with the exclusion of precursors 922.0098 m/z (z = 1) and 121.0509 (z = 1) set as reference ions. Data was extracted with MH+ mass range between 600-4000 Da and processed with Agilent Spectrum Mill MS Proteomics Workbench software packages. Carbamidomethylation of cysteine was set as a single modification. The peptide finger mapping was modified to specifically search against a merged database consisting of all non-redundant NCBI database with taxonomy set to Serpentes (taxid: 8570), and private database generated from the venom-gland transcriptome of the particular snake used in this study. Protein identifications were validated with the following filters: protein score >11, peptides score >6 and scored peak intensity (SPI) >60 %. Only results with “Distinct Peptide” identification of 2 or greater than 2 are considered significant.

### Gel intensity estimation and label-free LCMS/MS protein quantitation

The abundance of a protein within a gel section was determined based on the mean spectral intensity (MSI) relative to the total mean spectral intensity of all proteins from the gel section. Label-free LCMS/MS relative quantitation methods have been recently applied in many studies including venom shotgun proteomics [31]. To compensate for potential discrepancy between gel sections due to manual handling on gel excision and in-gel digestion, the relative MSI of a protein was adjusted according to the relative intensity of the corresponding gel section (determined with Thermo Scientific Pierce myImage Analysis Software). The generic equation is summarized below:

$$ \begin{array}{l} Relative\kern0.5em  abundance\kern0.5em  of\kern0.5em  protien\kern0.5em A\kern0.5em  in\kern0.5em  gel\kern0.5em  \sec tion\kern0.5em 1\kern0.5em =\kern0.5em \\ {}\left(\frac{Mean\kern0.5em  spectral\kern0.5em  in tensity\kern0.5em  of\kern0.5em  protien\kern0.5em A\kern0.5em  in\kern0.5em  Section\kern0.5em 1}{Total\kern0.5em  mean\kern0.5em  spectral\kern0.5em  in tensity\kern0.5em  in\kern0.5em  Section\kern0.5em 1}\right)\kern0.5em x\kern0.5em  Relative\kern0.5em  gel\kern0.5em  in tensity\kern0.5em  of\kern0.5em  Section\kern0.5em 1\kern0.5em \left(\%\right)\end{array} $$

The total abundance of a protein throughout the SDS-PAGE lane is the sum of its relative abundances from all gel sections.

### Correlation of mRNA expression and protein abundance

The method was modified from Aird et al. [31]; essentially the protein expression was measured based on peptide unit, analogous to the analysis for RNA expression described by [38]. In our study, for each protein identified in every gel section, the expression of its peptide fragment was first determined by dividing the number of its fragments with the number of total fragments in the gel section. The ratio is then multiplied with the corresponding gel section intensity (% by densitometry) to obtain the normalized measure of its peptide fragment per section (PFPS). The PFPS was then further divided by the length of the protein in order to normalize for size across various proteins, hence producing a measure of peptides per unit length (PPUL) of protein as described by Aird et al. [31]. The following is the calculation for the PPUL of a protein:

$$ \frac{\left(\frac{Peptide\kern0.5em  fragment\kern0.5em  in\kern0.5em  section}{Total\kern0.5em  peptide\kern0.5em  fragment\kern0.5em  in\kern0.5em  same\kern0.5em  section}\kern0.5em \times \kern0.5em  Relative\kern0.5em  gel\kern0.5em  in tensity\kern0.5em  of\kern0.5em  the\kern0.5em  section\right)}{Protein\kern0.5em  length} $$

Subsequently, the PPUL and FPKM values and the redundancies were cumulated according to protein family. Correlations were estimated by using parametric (Pearson’s product–moment correlation coefficients) statistics.

### Sequence alignment and phylogeny tree construction

Amino acid sequences form relevant comparative venom toxins were retrieved from the UniProtKB database (http://www.uniprot.org/). Multiple sequence alignment was performed with MUSCLE program [85] using Jalview software [86] and phylogenetic tree was constructed with Mega 6 [87] using the Maximum Likelihood method. FigTree 1.4 was used to produce phylogenetic tree figures. Neurotoxin-1 (P01479) of the Saharan scorpion *Androctonus australis* was used as outgroup.

### Availability of supporting data

Sequence data from the venom-gland transcriptome of the Malaysian king cobra, *Ophiophagus hannah* has been deposited in National Center for Biotechnology Information (NCBI) Sequence Read Archive (http://www.ncbi.nlm.nih.gov/Traces/sra/sra.cgi) under bioproject: PRJNA276622 (accession number SRP055563) (http://www.ncbi.nlm.nih.gov/sra/SRP055563).

The primary data (multiple sequence alignments and phylogenetic tree) and other additional files of data analyses have been deposited in Dryad Digital Repository (https://datadryad.org/resource/doi:10.5061/dryad.th547).

## Additional files

Additional file 1:
**Malaysian** ***Ophiophagus hannah*** **venom-gland** **transcriptome (Transcript FPKM >1).** (XLSX 13641 kb) Additional file 2:
**Malaysian** ***Ophiophagus hannah*** **Venom-gland** **Transcriptome Toxins Annotations.** (XLSX 147 kb) Additional file 3:
**Malaysian** ***Ophiophagus hannah*** **venom proteome.** (XLSX 223 kb) Additional file 4:
**Sequence comparision of Malaysian** ***Ophiophagus hannah*** **(MOh; current study) with Balinese species (BOh; Vonk et al. [** 20 **]) and other reported** ***O. hannah*** **toxins deposited in public database.** First column indicates the sources of the sequence used in the comparison. Second column indicates the protein family and ID of the current study and NCBI deposited public database (Abbrev.: 3FTx, three-finger toxin; SVMP, snake venom metalloproteinase; PLA2, phospholipase A2; CRISP, cysteine-rich secretory protein; KUN, Kunitz-type proteinase inhibitor; LAAO, L-amino acid oxidase; CVF, Cobra venom factor; IGF, insulin-like growth factor; NP, natriuretic peptide; SVSP, snake venom serine protease; PDE, phosphodiesterase; NGF, nerve growth factor; AChE, acetylcholinesterase; PLB, phospholipase-B; DPP IV, dipeptidylpeptidase IV; CTL, C-type lectin; VEGF, vascular endothelial growth factor). Third column shows the accession number of NCBI and SwissProt database on the first row and (where present) the number of the line in which the peptide match is found in the protein in the subsequent rows. Forth column shows the comparison of MOh with BOh (Vonk et al. [20]) and other reported *O. hannah* toxins using the sequences retrieved from MOh transcriptome (current study) and NCBI database. Fifth column shows the coverage of the MOh sequences as compared to the aligned sequences. Underline indicates signal peptide sequences; green indicates peptide matches; red indicates the amino acids diverse from the aligned sequences; purple indicated sequences neither matched to O. hannah species nor BOh toxins. *Sequence comparision were based on the annotated protein ID in Addtional file 2; two filters were made for the selections: 1) every protein ID annotated to *O. hannah* species; 2) proteins of other snake species which are also reported in Vonk et al. [20], i.e., neurotoxin-like protein 1, belonged to [Causus rhombeatus] species but reported in BOh species. (XLSX 31 kb)

## Abbreviations

MOh: Malaysian *Ophiophagus hannah*
3FTx: Three-finger toxin
SVMP: Snake venom metalloproteinase
PLA_2_: Phospholipase A_2_
CRISP: Cysteine-rich secretory protein
KUN: Kunitz-type proteinase inhibitor
LAAO: L-amino acid oxidase
CVF: Cobra venom factor
IGF: Insulin-like growth factor
NP: Natriuretic peptide
SVSP: Snake venom serine protease
PDE: Phosphodiesterase
NGF: Nerve growth factor
AChE: Acetylcholinesterase
PLB: Phospholipase-B
DPP IV: Dipeptidylpeptidase IV
CTL: C-type lectin
VEGF: Vascular endothelial growth factor
MSI: Mean spectral intensity
FPKM: Fragments per kilobase of exon model per million mapped reads

## References

[1] Fry BG, Vidal N, Norman JA, Vonk FJ, Scheib H, Ramjan SF, Kuruppu S, Fung K, Hedges SB, Richardson MK (2006). Early evolution of the venom system in lizards and snakes. Nature.

[2] Kordis D, Gubensek F (2000). Adaptive evolution of animal toxin multigene families. Gene.

[3] Casewell NR, Wuster W, Vonk FJ, Harrison RA, Fry BG (2013). Complex cocktails: the evolutionary novelty of venoms. Trends Ecol Evol.

[4] Calvete JJ, Sanz L, Angulo Y, Lomonte B, Gutierrez JM (2009). Venoms, venomics, antivenomics. FEBS Lett.

[5] Warrell DA. Guidelines for the management of snake-bites. World Health Organ. 2010.

[6] Jamaiah I, Rohela M, Ng TK, Ch'ng KB, Teh YS, Nurulhuda AL, Suhaili N (2006). Retrospective prevalence of snakebites from Hospital Kuala Lumpur (HKL) (1999-2003). Southeast Asian J Trop Med Public Health.

[7] Jamaiah I, Rohela M, Roshalina R, Undan RC (2004). Prevalence of snake bites in Kangar District Hospital, Perlis, west Malaysia: a retrospective study (January 1999-December 2000). Southeast Asian J Trop Med Public Health.

[8] Chew KS, Khor HW, Ahmad R, Rahman NH (2011). A five-year retrospective review of snakebite patients admitted to a tertiary university hospital in Malaysia. Int J Emerg Med.

[9] O'shea M. Venomous Snakes of the World. Princeton University Press, New Jersey. 160 pp.

[10] Tan NH, Saifuddin MN (1989). Isolation and characterization of an unusual form of L-amino acid oxidase from King cobra (Ophiophagus hannah) venom. Biochem Int.

[11] Guo XX, Zeng L, Lee WH, Zhang Y, Jin Y (2007). Isolation and cloning of a metalloproteinase from king cobra snake venom. Toxicon.

[12] He YY, Lee WH, Zhang Y (2004). Cloning and purification of alpha-neurotoxins from king cobra (Ophiophagus hannah). Toxicon.

[13] Li J, Zhang H, Liu J, Xu K (2006). Novel genes encoding six kinds of three-finger toxins in Ophiophagus hannah (king cobra) and function characterization of two recombinant long-chain neurotoxins. Biochem J.

[14] Tan NH, Saifuddin MN (1990). Purification and characterization of two acidic phospholipase A2 enzymes from king cobra (Ophiophagus hannah) snake venom. Int J Biochem.

[15] Wang QY, Shu YY, Zhuang MX, Lin ZJ (2001). Cloning and Sequence Analysis of cDNAs Encoding Two Acidic PLA(2) from venom of Ophiophagus hannah(King Cobra), Guangxi Species. Sheng Wu Hua Xue Yu Sheng Wu Wu Li Xue Bao (Shanghai).

[16] Pung YF, Wong PT, Kumar PP, Hodgson WC, Kini RM (2005). Ohanin, a novel protein from king cobra venom, induces hypolocomotion and hyperalgesia in mice. J Biol Chem.

[17] He YY, Liu SB, Lee WH, Qian JQ, Zhang Y (2008). Isolation, expression and characterization of a novel dual serine protease inhibitor, OH-TCI, from king cobra venom. Peptides.

[18] Lee WH, Zhang Y, Wang WY, Xiong YL, Gao R (1995). Isolation and properties of a blood coagulation factor X activator from the venom of king cobra (Ophiophagus hannah). Toxicon.

[19] Chang HC, Tsai TS, Tsai IH (2013). Functional proteomic approach to discover geographic variations of king cobra venoms from Southeast Asia and China. J Proteomics.

[20] Vonk FJ, Casewell NR, Henkel CV, Heimberg AM, Jansen HJ, McCleary RJ, Kerkkamp HM, Vos RA, Guerreiro I, Calvete JJ (2013). The king cobra genome reveals dynamic gene evolution and adaptation in the snake venom system. Proc Natl Acad Sci USA.

[21] Mackessy SP, Mackessy SP (2009). The field of reptile toxinology. Snakes, lizards, and their venoms. Handbook of venoms and toxins of reptiles.

[22] Gutierrez JM, Williams D, Fan HW, Warrell DA (2010). Snakebite envenoming from a global perspective: Towards an integrated approach. Toxicon.

[23] Williams DJ, Gutierrez JM, Calvete JJ, Wuster W, Ratanabanangkoon K, Paiva O, Brown NI, Casewell NR, Harrison RA, Rowley PD (2011). Ending the drought: new strategies for improving the flow of affordable, effective antivenoms in Asia and Africa. J Proteomics.

[24] Vejayan J, Khoon TL, Ibrahim H (2014). Comparative analysis of the venom proteome of four important Malaysian snake species. J Venom Anim Toxins Incl Trop Dis.

[25] Danpaiboon W, Reamtong O, Sookrung N, Seesuay W, Sakolvaree Y, Thanongsaksrikul J, Dong-din-on F, Srimanote P, Thueng-in K, Chaicumpa W (2014). Ophiophagus hannah venom: proteome, components bound by Naja kaouthia antivenin and neutralization by N. kaouthia neurotoxin-specific human ScFv. Toxins.

[26] Grabherr MG, Haas BJ, Yassour M, Levin JZ, Thompson DA, Amit I, Adiconis X, Fan L, Raychowdhury R, Zeng Q (2011). Full-length transcriptome assembly from RNA-Seq data without a reference genome. Nat biotechnol.

[27] Dyballa N, Metzger S. Fast and sensitive colloidal coomassie G-250 staining for proteins in polyacrylamide gels. J Vis Exp. 2009;30. 10.3791/1431 PMC3149902 19684561

[28] Kini RM, Doley R (2010). Structure, function and evolution of three-finger toxins: mini proteins with multiple targets. Toxicon.

[29] Barber CM, Isbister GK, Hodgson WC (2013). Alpha neurotoxins. Toxicon.

[30] Li S, Wang J, Zhang X, Ren Y, Wang N, Zhao K, Chen X, Zhao C, Li X, Shao J (2004). Proteomic characterization of two snake venoms: Naja naja atra and Agkistrodon halys. Biochem J.

[31] Aird SD, Watanabe Y, Villar-Briones A, Roy MC, Terada K, Mikheyev AS (2013). Quantitative high-throughput profiling of snake venom gland transcriptomes and proteomes (Ovophis okinavensis and Protobothrops flavoviridis). BMC Genomics.

[32] Calvete JJ (2014). Next-generation snake venomics: protein-locus resolution through venom proteome decomplexation. Expert Rev Proteomics.

[33] Tan NH (1991). The Biochemistry of venoms of some venomous snakes of Malaysia - A review. Trop Biomed.

[34] Yap MK, Fung SY, Tan KY, Tan NH (2014). Proteomic characterization of venom of the medically important Southeast Asian Naja sumatrana (Equatorial spitting cobra). Acta Trop.

[35] Takacs Z, Wilhelmsen KC, Sorota S (2004). Cobra (Naja spp.) nicotinic acetylcholine receptor exhibits resistance to Erabu sea snake (Laticauda semifasciata) short-chain alpha-neurotoxin. J Mol Evol.

[36] Vogel C, Marcotte EM (2012). Insights into the regulation of protein abundance from proteomic and transcriptomic analyses. Nat Rev Genet.

[37] Durban J, Juarez P, Angulo Y, Lomonte B, Flores-Diaz M, Alape-Giron A, Sasa M, Sanz L, Gutierrez JM, Dopazo J (2011). Profiling the venom gland transcriptomes of Costa Rican snakes by 454 pyrosequencing. BMC Genomics.

[38] Mortazavi A, Williams BA, McCue K, Schaeffer L, Wold B (2008). Mapping and quantifying mammalian transcriptomes by RNA-Seq. Nat Methods.

[39] Kini RM (2002). Molecular moulds with multiple missions: functional sites in three-finger toxins. Clin Exp Pharmacol Physiol.

[40] Tan KY, Tan CH, Fung SY, Tan NH (2015). Venomics, lethality and neutralization of Naja kaouthia (monocled cobra) venoms from three different geographical regions of Southeast Asia. J Proteomics.

[41] Hedge RP, Rajagopalan N, Doley R, Kini RM, Mackessy SP (2009). Snake venom three-finger toxins. Handbook of venoms and toxins of reptiles.

[42] Pahari S, Mackessy SP, Kini RM (2007). The venom gland transcriptome of the Desert Massasauga rattlesnake (Sistrurus catenatus edwardsii): towards an understanding of venom composition among advanced snakes (Superfamily Colubroidea). BMC Mol Biol.

[43] Rokyta DR, Lemmon AR, Margres MJ, Aronow K (2012). The venom-gland transcriptome of the eastern diamondback rattlesnake (Crotalus adamanteus). BMC Genomics.

[44] Pawlak J, Mackessy SP, Fry BG, Bhatia M, Mourier G, Fruchart-Gaillard C, Servent D, Menez R, Stura E, Menez A (2006). Denmotoxin, a three-finger toxin from the colubrid snake Boiga dendrophila (Mangrove Catsnake) with bird-specific activity. J Biol Chem.

[45] Antil S, Servent D, Menez A (1999). Variability among the sites by which curaremimetic toxins bind to torpedo acetylcholine receptor, as revealed by identification of the functional residues of alpha-cobratoxin. J Biol Chem.

[46] Fry BG, Wuster W, Kini RM, Brusic V, Khan A, Venkataraman D, Rooney AP (2003). Molecular evolution and phylogeny of elapid snake venom three-finger toxins. J Mol Evol.

[47] Pillet L, Tremeau O, Ducancel F, Drevet P, Zinn-Justin S, Pinkasfeld S, Boulain JC, Menez A (1993). Genetic engineering of snake toxins. Role of invariant residues in the structural and functional properties of a curaremimetic toxin, as probed by site-directed mutagenesis. J Biol Chem.

[48] Tremeau O, Lemaire C, Drevet P, Pinkasfeld S, Ducancel F, Boulain JC, Menez A (1995). Genetic engineering of snake toxins. The functional site of Erabutoxin a, as delineated by site-directed mutagenesis, includes variant residues. J Biol Chem.

[49] Leong PK, Sim SM, Fung SY, Sumana K, Sitprija V, Tan NH (2012). Cross neutralization of Afro-Asian cobra and Asian krait venoms by a Thai polyvalent snake antivenom (Neuro Polyvalent Snake Antivenom). PLoS Negl Trop Dis.

[50] Alagesh TN. Zookeeper in coma after cobra attack. The New Straits Times 2012, March 3: Last assessed on August 23, 2015: http://news.asiaone.com/News/AsiaOne+News/Malaysia/Story/A1Story20120304-331539.html.

[51] Tan NH, Hj MN (1989). Enzymatic and toxic properties of Ophiophagus hannah (king cobra) venom and venom fractions. Toxicon.

[52] Fox JW, Serrano SM (2005). Structural considerations of the snake venom metalloproteinases, key members of the M12 reprolysin family of metalloproteinases. Toxicon.

[53] Markland FS, Swenson S (2013). Snake venom metalloproteinases. Toxicon.

[54] Guan HH, Goh KS, Davamani F, Wu PL, Huang YW, Jeyakanthan J, Wu WG, Chen CJ (2010). Structures of two elapid snake venom metalloproteases with distinct activities highlight the disulfide patterns in the D domain of ADAMalysin family proteins. J Struct Biol.

[55] Yamakawa Y, Omori-Satoh T (1988). A protease in the venom of king cobra (Ophiophagus hannah): purification, characterization and substrate specificity on oxidized insulin B-chain. Toxicon.

[56] Tan NH, Saifuddin MN (1990). Isolation and characterization of a hemorrhagin from the venom of Ophiophagus hannah (king cobra). Toxicon.

[57] Weissenberg S, Ovadia M, Kochva E (1987). Species specific sensitivity towards the hemorrhagin of Ophiophagus hannah (Elapidae). Toxicon.

[58] Yamazaki Y, Hyodo F, Morita T (2003). Wide distribution of cysteine-rich secretory proteins in snake venoms: isolation and cloning of novel snake venom cysteine-rich secretory proteins. Arch Biochem Biophys.

[59] Pawelek PD, Cheah J, Coulombe R, Macheroux P, Ghisla S, Vrielink A (2000). The structure of L-amino acid oxidase reveals the substrate trajectory into an enantiomerically conserved active site. EMBO J.

[60] Tan NH, Fung SY, Mackessy SP (2009). Snake venom L-amino acid oxidase. Handbook of venoms and toxins of reptiles.

[61] Lee ML, Fung SY, Chung I, Pailoor J, Cheah SH, Tan NH (2014). King cobra (Ophiophagus hannah) venom L-amino acid oxidase induces apoptosis in PC-3 cells and suppresses PC-3 solid tumor growth in a tumor xenograft mouse model. Int J Med Sci.

[62] Pung YF, Kumar SV, Rajagopalan N, Fry BG, Kumar PP, Kini RM (2006). Ohanin, a novel protein from king cobra venom: its cDNA and genomic organization. Gene.

[63] Huang MZ, Gopalakrishnakone P, Chung MC, Kini RM (1997). Complete amino acid sequence of an acidic, cardiotoxic phospholipase A2 from the venom of Ophiophagus hannah (King Cobra): a novel cobra venom enzyme with "pancreatic loop". Arch Biochem Biophys.

[64] Zeng L, Sun QY, Jin Y, Zhang Y, Lee WH, Zhang Y (2012). Molecular cloning and characterization of a complement-depleting factor from king cobra, Ophiophagus hannah. Toxicon.

[65] Vogel CW, Fritzinger DC (2010). Cobra venom factor: Structure, function, and humanization for therapeutic complement depletion. Toxicon.

[66] Fry BG, Wuster W (2004). Assembling an arsenal: origin and evolution of the snake venom proteome inferred from phylogenetic analysis of toxin sequences. Mol Biol Evol.

[67] Turner AJ, Isaac RE, Coates D (2001). The neprilysin (NEP) family of zinc metalloendopeptidases: genomics and function. BioEssays.

[68] Casewell NR, Harrison RA, Wuster W, Wagstaff SC (2009). Comparative venom gland transcriptome surveys of the saw-scaled vipers (Viperidae: Echis) reveal substantial intra-family gene diversity and novel venom transcripts. BMC Genomics.

[69] Zhang Y, Lee WH, Xiong YL, Wang WY, Zu SW (1994). Characterization of OhS1, an arginine/lysine amidase from the venom of king cobra (Ophiophagus hannah). Toxicon.

[70] Tan CH, Tan NH, Sim SM, Fung SY, Gnanathasan CA (2015). Proteomic investigation of Sri Lankan hump-nosed pit viper (Hypnale hypnale) venom. Toxicon.

[71] Chatrath ST, Chapeaurouge A, Lin Q, Lim TK, Dunstan N, Mirtschin P, Kumar PP, Kini RM (2011). Identification of novel proteins from the venom of a cryptic snake Drysdalia coronoides by a combined transcriptomics and proteomics approach. J Proteome Res.

[72] Frobert Y, Creminon C, Cousin X, Remy MH, Chatel JM, Bon S, Bon C, Grassi J (1997). Acetylcholinesterases from Elapidae snake venoms: biochemical, immunological and enzymatic characterization. Biochim Biophys Acta.

[73] Ahmed MA, Rocha JB, Morsh VM, Schetinger MRC, Mackessy SP (2009). Snake venom acetylcholinesterases. Handbook of venoms and toxins of reptiles.

[74] Blesch A, Tuszynski MH (2004). Gene therapy and cell transplantation for Alzheimer's disease and spinal cord injury. Yonsei Med J.

[75] Koh DC, Armugam A, Jeyaseelan K (2006). Snake venom components and their applications in biomedicine. Cell Mol Life Sci.

[76] Aird SD (2002). Ophidian envenomation strategies and the role of purines. Toxicon.

[77] Dhananjaya BL, D'Souza CJ (2010). The pharmacological role of nucleotidases in snake venoms. Cell Biochem Funct.

[78] Aird SD, Mackessy SP (2009). The role of purine and pyrimidine nucleosides in snake venoms. Handbook of venoms and toxins of reptiles.

[79] Russell FE, Buess FW, Woo MY (1963). Zootoxicological properties of venom phosphodiesterase. Toxicon.

[80] Yap MK, Tan NH, Sim SM, Fung SY, Tan CH (2014). Pharmacokinetics of Naja sumatrana (Equatorial Spitting Cobra) Venom and Its Major Toxins in Experimentally Envenomed Rabbits. PLoS Negl Trop Dis.

[81] Rotenberg D, Bamberger ES, Kochva E (1971). Studies on ribonucleic acid synthesis in the venom glands of Vipera palaestinae (Ophidia, Reptilia). Biochem J.

[82] Iseli C, Jongeneel CV, Bucher P. ESTScan: a program for detecting, evaluating, and reconstructing potential coding regions in EST sequences. Proceedings / International Conference on Intelligent Systems for Molecular Biology; ISMB International Conference on Intelligent Systems for Molecular Biology; Heidelberg, 1999:138–148. 10786296

[83] Corpet F (1988). Multiple sequence alignment with hierarchical clustering. Nucleic Acids Res.

[84] Laemmli UK (1970). Cleavage of structural proteins during the assembly of the head of bacteriophage T4. Nature.

[85] Edgar RC (2004). MUSCLE: a multiple sequence alignment method with reduced time and space complexity. BMC Bioinforma.

[86] Waterhouse AM, Procter JB, Martin DM, Clamp M, Barton GJ (2009). Jalview Version 2--a multiple sequence alignment editor and analysis workbench. Bioinformatics.

[87] Tamura K, Stecher G, Peterson D, Filipski A, Kumar S (2013). MEGA6: Molecular Evolutionary Genetics Analysis version 6.0. Mol Biol Evol.

